# Artificially innervated self-healing foams as synthetic piezo-impedance sensor skins

**DOI:** 10.1038/s41467-020-19531-0

**Published:** 2020-11-12

**Authors:** Hongchen Guo, Yu Jun Tan, Ge Chen, Zifeng Wang, Glenys Jocelin Susanto, Hian Hian See, Zijie Yang, Zi Wei Lim, Le Yang, Benjamin C. K. Tee

**Affiliations:** 1 NUS Graduate School for Integrative Sciences and Engineering (NGS), National University of Singapore, Singapore, Singapore; 2 Department of Materials Science and Engineering (MSE), National University of Singapore, Singapore, Singapore; 3 Institute of Innovation in Health Technology (iHealthtech), National University of Singapore, Singapore, Singapore; 4 Institute of Materials Research and Engineering, Agency for Science Technology and Research, Singapore, Singapore; 5 Electrical and Computer Engineering, National University of Singapore, Singapore, Singapore; 6 N.1 Institute of Health, National University of Singapore, Singapore, Singapore

**Keywords:** Electrical and electronic engineering, Materials for devices, Soft materials

## Abstract

Human skin is a self-healing mechanosensory system that detects various mechanical contact forces efficiently through three-dimensional innervations. Here, we propose a biomimetic artificially innervated foam by embedding three-dimensional electrodes within a new low-modulus self-healing foam material. The foam material is synthesized from a one-step self-foaming process. By tuning the concentration of conductive metal particles in the foam at near-percolation, we demonstrate that it can operate as a piezo-impedance sensor in both piezoresistive and piezocapacitive sensing modes without the need for an encapsulation layer. The sensor is sensitive to an object’s contact force directions as well as to human proximity. Moreover, the foam material self-heals autonomously with immediate function restoration despite mechanical damage. It further recovers from mechanical bifurcations with gentle heating (70 °C). We anticipate that this material will be useful as damage robust human-machine interfaces.

## Abstract (web summary)

Designing mechanosensory system that detects mechanical contact forces like human skin remains a challenge. Here, the authors present artificially innervated self-healing foams by embedding 3D electrodes for piezo-impedance sensors that can operate in both piezoresistive and piezocapacitive sensing modes to address various proximity and mechanical interactions efficiently.

## Introduction

Human skin has remarkable self-healing abilities while being innervated by a wide variety of sensory neuron subtypes known as mechanoreceptors^1,2^. These mechanoreceptors buried beneath the skin extending into the epidermis convey the tactile stimuli to the nerves and enable us to use the sense of touch to manipulate objects, perform social communication, and react to unstructured external environments^1,2^. To mimic human skins, electronic skin (e-skins) has progressed swiftly from purely fictional imaginations to increasingly sophisticated embodiments over the last decade^3–7^. Such synthetic skins have great potential in a large variety of applications including health care, human–machine interactions, and robotics^3^. Although many different tactile sensing e-skins have been demonstrated^8–11^, an integrated e-skin sensor material that is self-healing, detects proximal precontact events and senses force directions simultaneously have yet to be demonstrated. There are excellent efforts to use ionic gels as artificial nerves in e-skins with organic transistors^12^, but the ionic gels based nerves did not show tactile sensitivity.

Furthermore, current e-skin tactile sensors have also relied heavily on either piezocapacitive or piezoresistive materials sandwiched between planar two-dimensional (2D) electrode patterns. The use of planar patterns with a sandwiched dielectric or conductive material could add complexity in the encapsulation assembly arising from potential air gaps or delamination. Moreover, the use of such 2D electrode patterns typically detects normal forces only. While there are reports on e-skins that detect both normal and shear forces^8,9,11,13–16^, most of them require encapsulation with sandwiched structures to work (Table 1). There are also designs of piezoresistive material pillars to achieve the normal and shear-force sensing, but these are not self-healing^17^.

**Table 1:** Comparison of different tactile sensor skins.

Sensors	Single substrate with single electrode layer	Sensing mechanism	Normal force	Shear force	Proximity sensing	Sensitivity (pressure range)	Self-healing	Reference
AiFoam	√	R & C	√	√	√	R: 0.0982 kPa^−1^ (*P* < 10 kPa) C: 0.378 kPa^−1^ (*P* < 10 kPa)	√	This work
Hill and micro-pyramidal structure	N	C	√	√	N	0.19 kPa^−1^ (*P* < 1 kPa)	N	^8^
Hairy skin electronics	N	R	√	√	N	0.25 kPa^−1^ (*P* < 100 kPa)	N	^11^
3D microelectromechanical sensor	N	R	√	√	N	~0.08 kPa^−1^ (*P* < 50 kPa)	N	^14^
Laminated structure with four electrodes	N	C	√	√	N	0.53 kPa^−1^ (0.5 < *P* < 2 kPa)	N	^15^
Bump on sensor	N	R	√	√	N	~0.0065 kPa^−1^ (*P* < 270 kPa)	N	^16^
Multifunctional matrix network	N	C	√	-	√	0.0224 kPa^−1^ (*P* < 16 kPa)	N	^10^

*N* no, √ yes, *R* resistive, *C* capacitive.

**Fig. 1:**
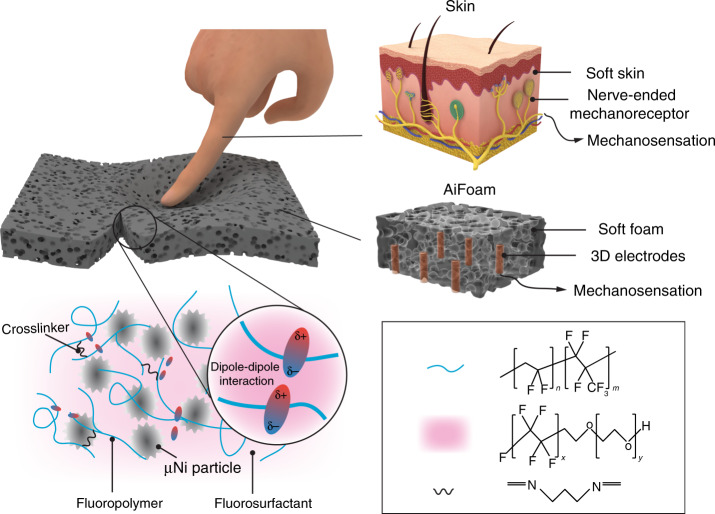
Artificially innervated pressure sensor that is low-modulus, elastic, and self-healing. *Left* The schematic diagram of the mechanism of the AiFoam material. The polymer chains (blue curves) are cross-linked by DAP (black curves) and surrounded by the fluorosurfactant (pink color) to form the low-modulus base elastomer. Micro-nickel (μNi) particles with surface nanostructure (gray particles) are mixed with the elastomer to form the foam material. The dipole–dipole interactions among the polymer chains and the surfactant molecule trap the surfactant within the polymer and enable the material to be self-healing. The AiFoam is fabricated by embedding three-dimensional electrodes in the foam material to mimic the innervations of the human skin. *Right* the schematic diagram of artificially innervated foam (AiFoam) sensor (*middle*) imitating the somatosensory innervation system of human skin (*top*).

Inspired by the architecture of the human somatosensory innervations, we propose a new structure that uses three-dimensional (3D) metal wire electrodes as “nerves” embedded within a low-modulus yet elastic self-healing foam we termed artificially innervated foam (AiFoam) (Fig. 1). Compared with other foam-based sensors (Table 2), our synthesized self-healing foam material has a low-modulus of 600 kPa and is relatively elastic to provide a restoring force for contact forces sensing. The 3D electrodes in the sensing material enable the AiFoam to be sensitive to both normal and shear forces. By embedding the 3D electrodes within our unique near-percolation metal particle foam composite, we show that our sensor can operate in both piezoresistive and piezocapacitive modes. The AiFoam e-skin can detect both tactile contact and proximity of the human touch.

**Table 2:** Comparison of foam sensing materials.

Foam material	Tactile sensing mechanism	Proximity sensing	Sensitivity (pressure range)	Self-healing	Reference
Cross-linked PVDF-HFP/fluorosurfactant foam	R & C	√	R: 0.0982 kPa^−1^ (*P* < 10 kPa) C: 0.378 kPa^−1^ (*P* < 10 kPa)	√	This work
MWNT-rGO-wrapped PU foam	R	N	0.088 kPa^−1^ (2.7 < *P* < 10 kPa)	N	^30^
Ni-foam-templated BNF@PDMS foam	C	N	0.854 kPa^−1^ (0 < *P* < 0.5 kPa)	N	^22^
CB@PU sponge	R	N	0.068 kPa^−1^ (0 < *P* < 2.5 kPa)	N	^31^
PANIH/C-RGO@PU sponge	R	N	0.0109 kPa^−1^ (12 < *P* < 25 kPa)	N	^32^
Sugar-templated CNT-PDMS sponge	R	N	0.03 kPa^−1^ (0 < *P* < 15 kPa)	N	^21^

*N* no, √ yes, *R* resistive, *C* capacitive, *MWNT* multiwalled carbon nanotube, *rGO* reduced graphene oxide, *PU* polyurethane, *BNF* boron nitride foam, *PDMS* polydimethylsiloxane, *CB* carbon black, *PANIH* polyaniline nanohair, *C-RGO* microcracked reduced graphene oxide, *CNT* carbon nanotube.

## Results

### Artificial innervation with 3D electrodes

**Fig. 2:**
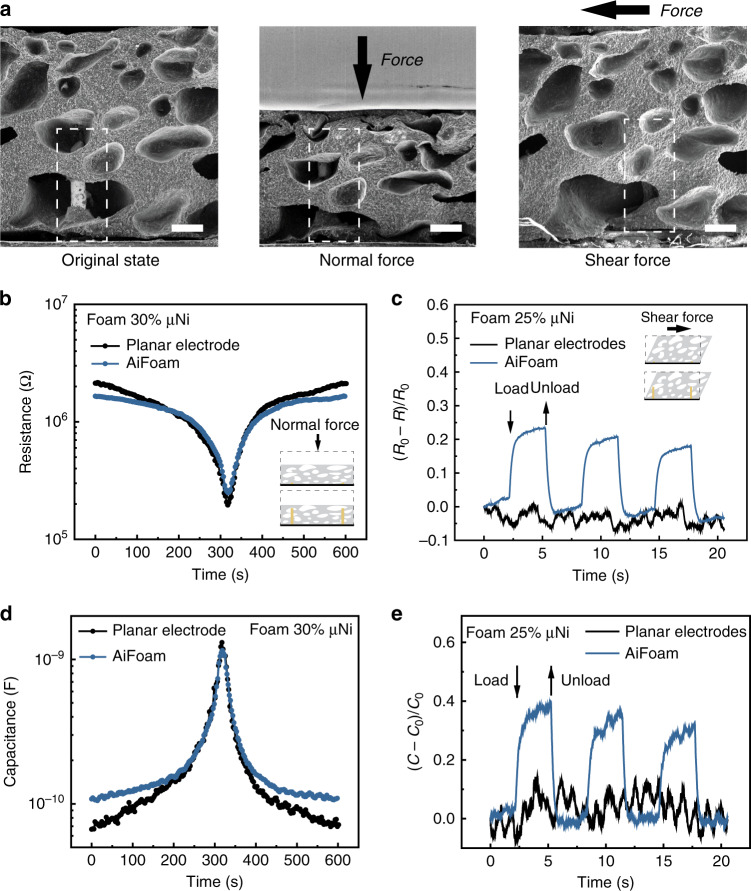
Artificial innervation for normal and shear-force detection of AiFoam. **a** SEM images of AiFoam sensor with 3D electrodes, revealing the original state (*left*), normal-force loading state (*middle*) and shear-force loading state (*right*), respectively. The black arrows represent the applied force directions. The dotted white boxes show the relative deformation of foam material to the 3D copper electrode. Scale bar: 200 μm. **b** Resistance and **d** capacitance responses on applying the normal force of 15 N to the Aifoam (with 30 vol% μNi) and control with 2D planar electrodes. Note that the data of **b** and **d** were simultaneously collected. **c** Resistance change and **e** capacitance change responses on applying the shear force to the AiFoam (with 25 vol% μNi) and control with 2D planar electrodes. Note that the data of **c** and **e** were simultaneously collected. *R*_0_ and *C*_0_ represent the initial resistance and capacitance of the sensor, whereas *R* and *C* represent the resistance and capacitance of the sensor during the pressure test.

Figure 2a shows the images of AiFoam with 3D electrodes embedded. When a normal or shear-force is applied, the foam material will be compressed, which deforms in shape to the relative electrodes. This deformation causes a measurable electrical impedance change. The 3D electrodes are flexible copper wires with a radius of 75 μm soldered on a flexible printed circuit board (PCB). The polymer was then cast on the flexible PCB, inducing self-foaming by evaporation-induced phase inversion. The 3D electrodes hold the foam material in place, even if the sensing material is delaminated from the PCB due to wear-and-tear (Supplementary Fig. 1).

We compared our AiFoam with 3D electrodes (AiFoam) to a similarly prepared control foam sensor with planar electrodes (Foam-2D) by applying normal and shear forces. The piezoresistance effect decreases electrical resistance of both electrode configurations when an external pressure was applied (Fig. 2b), but AiFoam exhibited lower initial resistance because of the larger contact areas of the 3D electrodes to the foam compared to the Foam-2D version. The extension of the 3D electrodes into the foam material enables more sensitive deformation sensing near the top surface compared to the planar electrodes that barely detected the deformations. Hence, when a shear force was applied on the top surface, AiFoam showed an obvious resistance drop (Fig. 2c) but Foam-2D device did not show consistently detectable resistance change. Thus, AiFoam can capture and respond to small changes in surface deformations from an external mechanical stimulus.

We found that we can operate AiFoam in both piezoresistive and piezocapacitive modes simultaneously due to the interfacial impedance of the foam material (Supplementary Fig. 2), which is partly capacitive as well as resistive in nature^18^. AiFoam can also detect normal and shear forces by monitoring capacitance changes. In piezocapacitive mode, the capacitance change increases when an external force was applied (Fig. 2d). The capacitance of the sensor also increased with the shear forces (Fig. 2e).

### Low-modulus, elastic, and self-healing foam

**Fig. 3:**
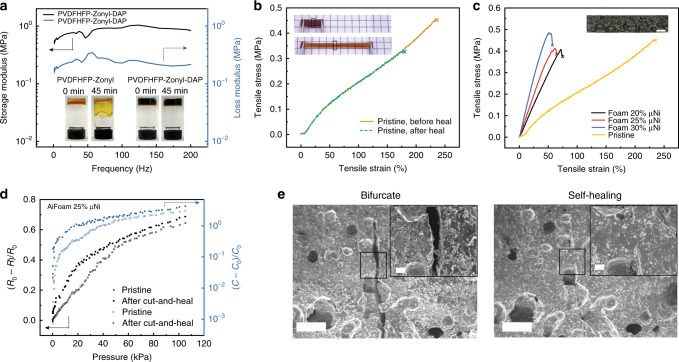
Characterization of a low-modulus, elastic, and self-healing foam material. **a** DMA result of the base polymer showing its viscoelasticity. PVDF-HFP-Zonyl represents the uncrosslinked material and PVDF-HFP-Zonyl-DAP represents the cross-linked material. Left insets (PVDF-HFP-Zonyl, 0 and 45 min) show the flowability of the material before crosslink. Right insets (PVDF-HFP-Zonyl-DAP, 0 and 45 min) show the solid-state material after crosslink. **b** Typical stress–strain curves of original and healed base elastomer material. The sample is bifurcated and then self-healed at 70 °C for 4 days. Top inset shows the self-healed sample at the original state. Bottom inset shows the self-healed sample being stretched. The dotted black boxes show the bifurcated and heal region on the sample. **c** Stress–strain curves of base elastomer and foam materials with different volume fractional μNi loadings. Inset: An optical image of the foam structure. Scale bar: 1 mm. **d** Graph shows the self-healing of piezoresistive and piezocapacitive behaviors of AiFoam (with 25 vol% μNi). *R*_0_ and *C*_0_ represent the initial resistance and capacitance of the sensor, whereas *R* and *C* represent the resistance and capacitance of the sensor during the pressure test. The data were simultaneously collected. **e** Cross-sectional SEM images reveal the self-healing performance of foam material; *left* bifurcated; *right* self-healed at 70 °C for 4 days. Insets: The zoom-in SEM images of the black boxes. Scale bar: 50 μm.

The base elastomer of the AiFoam is synthesized by mixing a fluoropolymer (poly(vinylidene fluoride)-co-hexafluoropropylene, PVDF-HFP) and a fluorosurfactant (Zonyl FS-300), followed by partial crosslinking of the mixture using 1,3-diaminopropane (DAP) (see “Methods”). Mixing at a mass ratio of 49:50 (polymer:fluorosurfactant) at 70 °C resulted in a flowable, gel-like polymer solution (Fig. 3a inset and Supplementary Fig. 3a) because the fluorosurfactant is a polar solvent of PVDF-HFP. When DAP was added and the crosslinking process was completed, the polymer solution becomes the base elastomer (Fig. 3a inset and Supplementary Fig. 3b).

We applied dynamic mechanical analysis (DMA) on the cross-linked base elastomer to determine its mechanical properties. The cross-linked base elastomer shows a higher storage modulus than loss modulus, indicating that our base elastomer is viscoelastic with a loss factor of 15°–19° within a frequency range of 1–200 Hz at ambient temperature. The viscoelasticity can be attributed to the DAP molecules that bridge the PVDF-HFP polymer chains (Supplementary Figs. 4, 5). The cross-linked elastomer was stable when tested to a temperature of 385 °C (Supplementary Fig. 6a–c).

The strong dipole–dipole interactions between the surfactant molecules and the cross-linked polymer network trap the surfactant strongly within the base elastomer. This imbued the elastomer with self-healing properties and enhanced dielectric permittivity^7^. When the surfactant was added, the glass transition temperature (*T*_g_) of our base elastomer decreased to −38.5 °C, while the virgin PVDF-HFP had a higher *T*_g_ of −21.8 °C (Supplementary Fig. 6d). This indicated the plasticizer function of the surfactant, which lowered the mechanical modulus of the resultant elastomer. The fluorosurfactant also contributed to the self-healing behavior of the elastomer via the dipole–dipole interaction and hydrogen bonding among the fluorinated terminals of the fluorosurfactant molecules and PVDF-HFP chains^7^. When a non-fluorinated surfactant was used, we found that the self-healing property of the polymer disappeared (Supplementary Fig. 7).

Our base elastomer has as low as a modulus of 310 kPa and can reach a strain of ~230%. After bifurcation, the cut interfaces self-healed at 70 °C after 4 days and recovered 76.3% of its maximum strain with a toughness healing efficiency of 63.2% ± 14.6% (Fig. 3b, Supplementary Table 1, Supplementary Movie 1). After self-healing, the elastomer can be strained up to 180% (Fig. 3b inset).

To make a piezoresistive elastomeric composite, we added micro-nickel (μNi) particles with nanoscale surface features as conductive fillers^19^. However, we discovered that this addition of particles resulted in a closed-cell foam material (Supplementary Fig. 8). Fortuitously, the porous structure enabled greater deformation when mechanical stress is applied without affecting the integrity of the 3D electrodes. The pore structure, size, and distribution were consistent across batches (Supplementary Fig. 9). Pore sizes are larger (~1 mm) near the substrate surface while smaller pores (0.13 mm) were found at the top of the foam.

The pore distribution can be explained by the void formation theory^20^, similar to the swiss cheese eyes formation (Supplementary Fig. 10). As the solvent evaporates from the polymer, the μNi particles (and Cu electrodes) served as void nucleation sites. Upon heating to 70 °C, evaporation-induced phase inversion causes greater void nucleation and aggregation, thereby generating pores within the polymer matrix. The bottom part of the polymer dries slower than the air-exposed top surface, resulting in voids aggregation that created larger pores. In contrast, when we evaporated the solvents very slowly at a low temperature, which prevented void nucleation, we found that no pores were formed, further validating our proposed pore formation mechanism. Hence, our foam material can be synthesized in a straightforward self-foaming process. Moreover, the porosity of foam materials and the sensor performance are consistent across batches (Supplementary Fig. 11).

This self-foaming method is convenient and scalable, compared to conventional processes that use sacrificial templates, such as sugar^21^, metal foam^22^, and PS beads^23^, or by using multiphase reaction^24^. Although these methods can produce small and uniformly distributed pores, the fabrication processes typically involve multiple steps and may still contain residues of the template materials.

We next studied foam materials with μNi concentrations of 20 volume percent (vol%), 25 vol% and 30 vol% to the base elastomer (see “Method”), which we refer to from now as Foam 20 μNi, Foam 25 μNi, and Foam 30 μNi, respectively. The ultimate strength of foam materials decreased only slightly compared with the base elastomer, which was remarkable considering pores were introduced into the material (Fig. 3c, Supplementary Fig. 12). However, the Young’s modulus of the foam material increased compared to the base elastomer (Supplementary Table 2). The addition of μNi increases the stiffness of the material and counteracts the introduction of pores that decreases the ultimate strength and maximum elongation.

Our metal-foam composite also exhibited self-healing properties (Fig. 3d, e). Damaged parts show diminished scars over time (Fig. 3e). The resistance of the composite material and its pressure sensing performance recovered almost immediately upon bifurcation and contact (Supplementary Fig. 13a, b). The piezoresistive and piezocapacitive behaviors of the AiFoam were also characterized after bifurcation and self-healing for 4 days at 70 °C (Fig. 3d and Supplementary Fig. 13c, d).

### Piezo-impedance tactile sensing

**Fig. 4:**
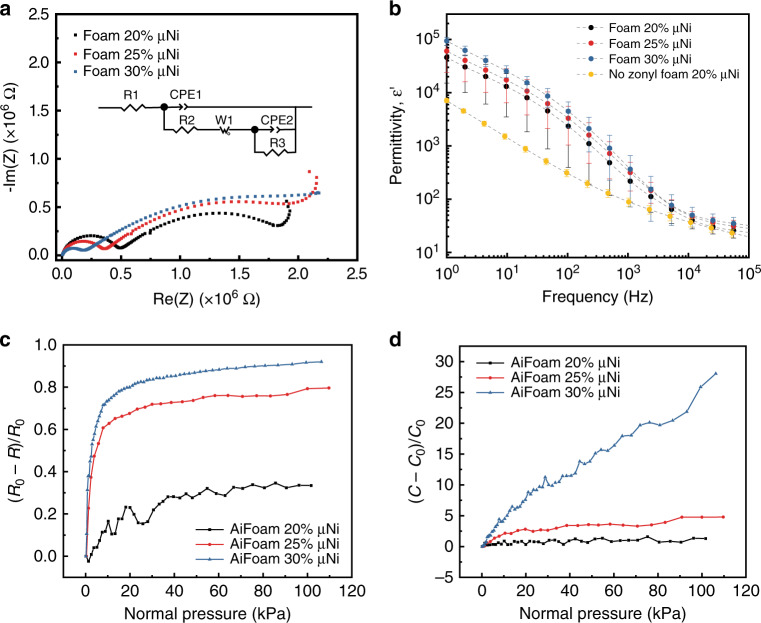
Electrical performance of AiFoam. **a** Nyquist plots of foam materials with different volume fractional μNi loadings. Re(Z) represents the real part of the impedance and Im(Z) represents the imaginary part of the impedance. Inset is the equivalent circuit of the foam materials, where *R*_1_ represents the bulk resistance; CPE_1_ and *R*_2_ represent the double-layered constant phase element (CPE) and the polarization resistance of one of the component, respectively; CPE_2_ and *R*_3_ represent the double-layered capacitance and the polarization resistance of the other component; *W*_1_ represents the mass transport process. CPE is used to account imperfect capacitance caused by imperfect interfaces or nonuniformity (Supplementary Fig. 17). **b** The real part of the dielectric permittivity of foam materials with different volume fractional μNi loadings under the frequency range of 1–10^5^ Hz. The error bars are calculated based on three samples. **c** Resistance and **d** capacitance change responses of AiFoam with different volume fractional μNi loadings under normal pressure. *R*_0_ and *C*_0_ represent the initial resistance and capacitance of the sensor, whereas *R* and *C* represent the resistance and capacitance of the sensor during the pressing test. The data of **c**, **d** were collected simultaneously.

The AiFoam can be operated as a piezo-impedance sensor with either capacitive or resistive changes measured. To better understand the electrical impedance characteristics of the metal-foam composites, we performed electrical impedance measurements. Nyquist plots of shows that Foam 20 μNi, Foam 25 μNi, and Foam 30 μNi decreased in baseline electrical resistance from 0.49 MOhm, 0.35 MOhm to 0.18 MOhm, respectively (Fig. 4a). The lowered electrical resistance of µNi foam is due to the nanostructured µNi particles dispersed throughout the elastomer matrix. These nanostructures are known to enhance the local electric fields^19^. As more µNi particles are incorporated into the elastomer matrix, percolation pathways for electrons emerge. This leads to a decrease in electrical resistance as more μNi particles were added (Supplementary Figs. 14–16).

The equivalent circuits (EC) of the sample were fitted from the impedance data (Fig. 4a inset and Supplementary Fig. 17). The capacitive behavior of the materials likely originated from the incorporation of fluorosurfactant. The Nyquist plot of the materials without fluorosurfactant (Supplementary Fig. 18a, b) showed the disappearance of a capacitive loop at a high frequency range. We found that the surfactant was significant for the electrical properties of the composite. A higher concentration of the surfactant can cause a more significant impedance decrease and a large shift of phase angles (Supplementary Fig. 18c, d). The highly asymmetric characteristics of the surfactant molecules, containing both C–F groups and ethoxyl groups, causes the adsorption/desorption interactions of the molecules with the various interfaces in the composite. Hence, it is hypothesized that the interactions between the surfactant and the neighboring materials (PVDF-HFP, uNi particles, and electrodes), are the primary causes of the capacitive loops. Pores inside the composites may also contribute to the capacitive loops (Supplementary Figs. 18e, f, 19).

We measured the dielectric permittivity of the foam composite materials in the frequency range of 0–10^5^ Hz (Fig. 4b). The samples have large permittivity values because of the dispersed fluorosurfactant and μNi fillers in the material matrix, as shown in the energy dispersive spectroscopy (EDS) mapping (Supplementary Fig. 20). Fluorosurfactant molecules improve the permittivity in the material via the realignment of dipole centers^7^. Samples with no surfactant showed ~8.5 times lower permittivity comparing to materials with surfactant (Fig. 4b).

Remarkably, Foam 30 μNi has a high permittivity of ~94,000 at a frequency of 1 Hz. This is because the μNi particles form conducting paths across the matrix of the material. Maxwell–Wagner effect induces high dielectric constant of the near-percolation composites at low frequencies^25,26^. As frequency increases, frequency-dependent relaxation causes a decrease in the dielectric permittivity of the composites^7,25^. At 1 kHz, the permittivity of Foam 30 μNi remains high at 370, and can be used in a large array of piezocapacitive sensors where high baseline capacitances are desired because capacitance scales linearly with electrode area. We found that nonporous samples have slightly higher permittivity than the foam samples (Supplementary Fig. 21) from the higher volume concentration of μNi.

The balance between the conductivity and dielectric permittivity provides the opportunity for using piezo-impedances (resistance and capacitance) operation in tactile sensing. When pressure (*P*) < 10 kPa, the sensitivity of AiFoam increases from 11.4 to 98.2 MPa^−1^ as μNi loadings increasing from 20 to 30 vol% (Fig. 4c). For AiFoam with 30 vol% μNi, the sensitivity was 1.25 MPa^−1^ when 10 kPa < *P* < 100 kPa. In piezocapacitive mode, the sensitivity shows a similar trend as the piezoresistive behavior with higher sensitivities (Fig. 4d). When *P* < 10 kPa, the sensitivity reached 378 MPa^−1^ for AiFoam with 30 vol% μNi and remained at 218 MPa^−1^ when 10 kPa < *P* < 100 kPa (Supplementary Fig. 22). The sensor response time was as fast as 19 ms (Supplementary Fig. 23), which is comparable to other works^24,27,28^. The sensor can also restore its initial states after its exposure to different temperatures and humidity levels (Supplementary Figs. 24, 25).

### Mechanical force distribution

**Fig. 5:**
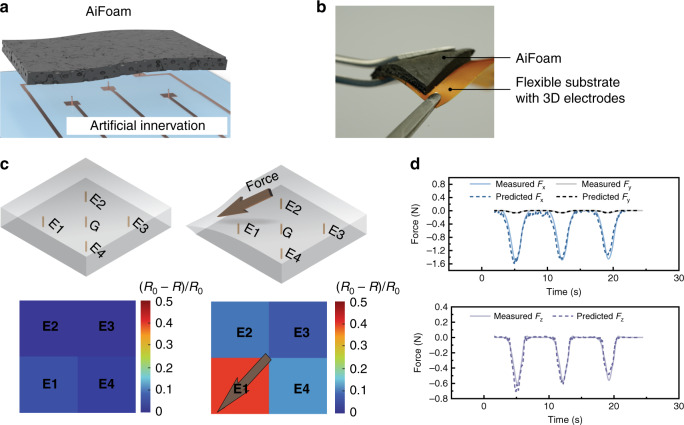
Direction identification. **a** Schematic and **b** photograph of AiFoam for direction identification. **c** AiFoam (with 30 vol% μNi) in detecting the direction and force distribution. *Top* Illustration of the deformation of a pixelated AiFoam sensor before (left) and under (right) external force loading; *bottom* resistance change response of AiFoam to the external force loadings. E1–E4 represents the four working electrodes and G represents the ground electrode. *R*_0_ represents the initial resistance of the sensor and *R* represents the resistance of the sensor during the pressing test. **d** Measured and predicted forces along different directions based on the result of **c**. *Top* Shear-force components along *x* and *y* directions (*F*_x_ and *F*_y_ respectively); *bottom* normal-force components along *z*-direction (*F*_z_).

We further developed an AiFoam sensor that could identify force distribution and direction by embedding four 3D electrodes and one ground electrode within the foam matrix (see “Methods”). The as-prepared AiFoam sensor is self-encapsulated and flexible (Fig. 5a, b). In this manner, we obtained four sensing zones: E1, E2, E3, and E4. When both normal and shear forces were applied simultaneously, the differential signals from the sensors allow us to determine the general direction of the applied force (Fig. 5c). For example, when a diagonal force was applied to E1 direction, E1 showed the largest resistance change while the other three zones have smaller resistance changes (Supplementary Movie 2).

In another example, we measured the resistance change when the force was applied in between E2 and E3 (Supplementary Fig. 26). To estimate the applied force vector, calibration is first conducted on the AiFoam (see “Methods”)^29^. The calibration of the AiFoam is based on the assumption that the calibration of a pair of electrodes (e.g., E1 and G) can also be used for the rest, since all electrode pairs are identical and independent of each other. Our estimated shear (*F*_x_ and *F*_y_) and normal forces (*F*_z_) showed good accuracy comparing with the measured forces (Fig. 5d). The accuracy between the peak force value of the predicted and measured forces are (95 ± 3)%, (98 ± 1)%, and (88 ± 6)% for *F*_x_, *F*_y_, and *F*_z_, respectively. Although only diagonal directions were applied in the demonstration, it can be extended to all directions.

### Proximity and tactile pressure sensing

**Fig. 6:**
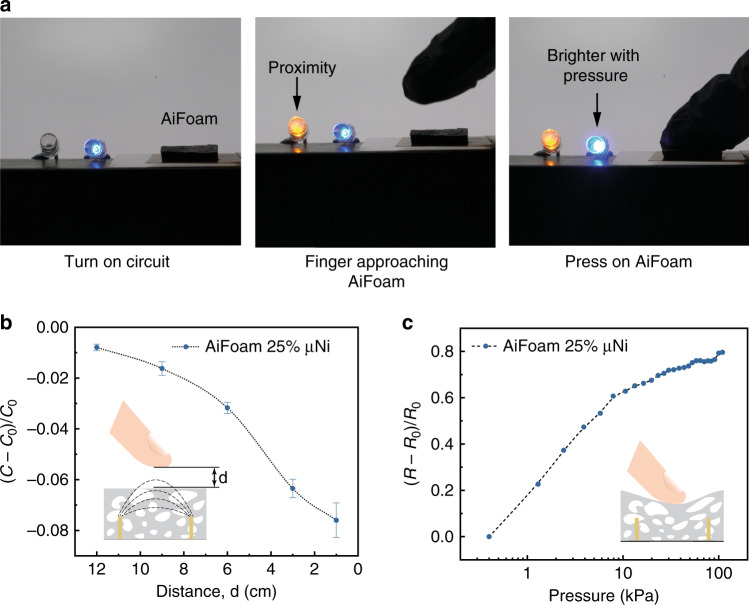
Capacitive proximity sensing and resistive pressure sensing. **a** Photographs showing both proximity sensing and pressure sensing of AiFoam sensor. *Left* The blue LED is activated, indicating the electrical circuit is turned on. *Middle* The orange LED lighted up when the human finger approaches to the sensor. *Right* The blue LED becomes brighter when the finger touches and presses the sensor. **b** The capacitance change responses on the distance between the finger and the AiFoam sensor (with 25 vol% μNi). The error bars are calculated based on three tests. *C*_0_ represents the initial capacitance of the sensor and *C* represents the capacitance of the sensor during the proximity test. *d* represents the distance between the human finger and AiFoam. **c** The resistance change responses on external pressure of AiFoam sensor (with 25 vol% μNi). *R*_0_ represents the initial resistance of the sensor and *R* represents the resistance of the sensor during the pressure test.

Intriguingly, AiFoam also can be used for proximity sensing. The fluorosurfactant and near-percolation μNi particles provide high dielectric permittivity values, enabling AiFoam to function well as a capacitive proximity sensor where the electrical field changes from the electrodes can be detected (Fig. 6a and Supplementary Movie 3). In contrast, a control sample of 25 vol% Ag nanoflakes (Ag Nfs) (see “Methods” and Supplementary Fig. 27) with high conductivity is not suitable for the proximity sensor (Supplementary Fig. 28). We measured the effect on the sensor capacitance versus the distance of the human finger to the AiFoam (Fig. 6b and Supplementary Fig. 29). The capacitance decreases as a human finger moves towards the sensor because the electric fields couple with the finger and reduces the effective capacitance between the 3D electrodes. When the finger contacts AiFoam and pressure was applied, the blue LED increased in intensity (Supplementary Movie 4) as resistance decreased with applied pressure (Fig. 6c). We demonstrated a simple circuit whereby both capacitance and resistance signal can be used for proximity sensing and pressure sensing, respectively (Supplementary Fig. 30).

## Discussion

Inspired by the innervated human mechanosensory system, we propose a self-healing artificially innervated foam (AiFoam) piezo-impedance tactile sensor using a new elastomeric polymer with metal particle composites. We show that innervating the foam material with embedded 3D electrodes, we can operate the tactile sensor in piezoresistive or piezocapacitive modes. The AiFoam sensor can differentiate between various force directions. Furthermore, it can function as a proximity sensor in capacitive mode, enabling smarter human–machine interactions in emerging augmented reality and robotic skin applications.

## Methods

### Fabrication of foam material

Fluorosurfactant (Zonyl FS-300) was dried in an oven at 70 °C and other chemicals were used as purchased. Two gram of poly(vinylidene fluoride)-co-hexafluoropropylene (PVDF-HFP) (3M) was dissolved in acetone and stirred for at least 4 h. 1.7 ml of fluorosurfactant was added to the solution and stirred for 24 h. Forty-six microliters of 1,3-diaminopropane (DAP) was added dropwise to the stirring solution. After 30 min, the solution turned light yellow. μNi microparticles were added to the solution and the mixture was mixed using a SpeedMixer (FlackTek) at 2500 rpm for 2.5 min. The mixture was cast into a 2.5 × 5 × 0.2 cm^3^ glass mold (width × length × height). The volume fraction of μNi particles in the polymer was calculated based on PVDF-HFP (Supplementary Table 6). The material was then heated on a hotplate at 70 °C for 30 min to self-foaming by evaporating acetone. To crosslink the polymer, the material was heated to 120 °C for 30 min.

### Fabrication of control material

Control sample with Ag Nanoflakes (Ag Nfs) was prepared by following the same procedures as above but changing μNi to Ag Nfs. The control samples of μNi-base elastomer composite with no pores were prepared by cooling the μNi, PVDF-HFP, fluorosurfactant, and DAP mixture for slow solvent evaporation before crosslinking.

### Fabrication of 3D electrode patterns

The electrode patterns on the flexible PCB were designed and etched (HCl:H_2_O_2_ = 1:3 (v/v)). The 3D electrodes were prepared by soldering copper wire with a radius of 75 μm on the designed planar point electrodes. The height of the 3D electrodes was ~1.2 mm. The rest of the planar electrode pattern was insulated by Kapton tape (3M). Copper wires were soldered to the flexible PCB to electrically connect it with the external power source.

### Electrical characterization

Samples with different μNi concentrations were prepared and cut into 1 × 1 cm^2^ size and sandwiched by two gold-sputtered foils. Then the samples were connected to an LCR meter (MFIA) for impedance analysis (*R* and *C* connected in series). The bulk resistance of the materials was fitted from the Nyquist plot using the Z-view software from the intercept on the real-axis at a high frequency. To characterize the I–V curves, 1 × 1 cm^2^ samples were placed on gold interdigital electrodes and connected to Keithley 2450. I–V characteristics were also characterized using a motorized *z*-axis stage (Newmark Systems). Force gauge (Newmark Systems) was used to apply loads to the sensors.

### Material characterization

The samples were imaged using an optical microscope (KEYENCE digital microscope) or an SEM (Zeiss sigma 300 with SmartEDS system). Mechanical testing was performed using an Instron Microtester 5500 instrument, testing at a rate of 1 mm s^−1^. The samples for the tensile test were cut into dumbbell shape following ASTM D1708. Self-healing experiments were performed at room temperature or 70 °C after bifurcating the samples and contact the cut regions for healing. Differential scanning calorimetry measurements from −70 to 200 °C with a heating speed of 20 °C/min were performed on TA Instruments DSC 25. Thermogravimetry analysis is done from 40 to 600 °C, with a heating rate of 20 °C on TA Instruments TGA Q500. The dielectric permittivity of the samples was measured using the Alpha-A high-performance frequency analyser (Novocontrol Technologies). FTIR-ATR measurements were performed on a VERTEX 70 spectrometer (Bruker) from 400 to 4000 cm^−1^.

### Normal-force detection

The pressure sensors were fabricated by directly casting polymer onto the 3D electrodes. The sizes of the sensors for normal-force detection were 1 × 1 cm^2^. The sensor was characterized using a motorized *z*-axis stage (Newmark Systems). Force gauge (Newmark Systems) was used to apply loads to the sensors covered with a piece of 1.5 × 1.5 cm^2^ glass slide on a custom-built probe station, all interfaced through a computer. For the sensor performance at different temperature and humidity values, the AiFoam sensor was tested in a temperature and humidity chamber (ESPECT). Weight was loaded (~5.4 kPa) on the AiFoam sensor to test the sensor performance at all the tested conditions. All the impedance signals (including absolute impedance, resistance, capacitance, and phase) were collected from the LCR meter simultaneously.

### Shear-force detection

The sensors for shear-force detection were fabricated by direct casting as mentioned above. A piece of 1.5 × 1.5 cm^2^ glass slide was cast on top of freshly cast material and cured together for a firm attachment. The setup of the shear-force test could be found in Supplementary Fig. 31. External forces were applied by pushing the glass slide sideways via a motorized *x*–*y*-axis stage (Newmark Systems). All the impedance signals (including absolute impedance, resistance, capacitance, and phase) were collected from the LCR meter simultaneously.

### Direction identification

The sensors for direction identification were fabricated by direct casting as mentioned above. The electrode patterns were designed with four pairs of electrodes as shown in Fig. 5a. L-shaped acrylic pieces were adhered on top of the sensor by a cyanoacrylate adhesive (Supplementary Fig. 26a). External forces were applied by pushing the L-shape acrylic via a motorized *x*–*y*-axis stage (Newmark Systems). Signals from the electrodes (E1, E2, E3, and E4) and the ground wire were collected via an LCR meter. All the impedance signals (including absolute impedance, resistance, capacitance, and phase) were collected from the LCR meter simultaneously.

### Force vector estimation

A specific calibration setup (Supplementary Fig. 32) has been prepared to acquire the applied force vector and the resistance change measured by a 3D electrode pair (i.e., E1 and G). This is made possible by mounting the AiFoam on a six-axis load cell (ATI Industrial Automation) and adhering an L-shaped acrylic structure on top of the AiFoam. The calibration of the AiFoam is carried out by applying a force onto the L-shaped structure in the direction towards E1. In particular, the force is applied carefully with a motorized *x*–*y*-axis stage (Newmark Systems), and all resistance variations and force components were measured by the LCR meter and load cell, respectively. These data are recorded at a sampling rate of 40 Hz and a moving average filter is applied. With the dataset, we formed calibration curves, and the accuracy of the calibration validated.

### Proximity demonstration

The sensor for proximity demonstration was fabricated by using AiFoam, connected to an LCR meter. Signals were collected when a human finger approached the sensor. For LED demonstration, the AiFoam sensor was fabricated on a 4-electrode pattern. Two of the electrodes were connected to an orange LED while the other two were connected to a blue LED (Supplementary Fig. 30). A DC power source was applied to activate the circuit.

## Supplementary information

Supplementary Information

Description of Additional Supplementary Files

Supplementary movie 1. Stretchability and self-healing ability of AiFoam elastomer

Supplementary movie 2. Direction sensing

Supplementary movie 3. Proximity sensing

Supplementary movie 4. Proximity and pressure sensing

## Data Availability

The data that support the findings in this study and the Supplementary Information are available from the corresponding author upon reasonable request.
