# Supplementary material for: Artificially innervated self-healing foams as synthetic piezo-impedance sensor skins
Source: Nat Commun. 2020 Nov 12;11:5747. doi: 10.1038/s41467-020-19531-0 (PMC7665015; doi:10.1038/s41467-020-19531-0)
Supplement: Supplementary file 1 — Supplementary Information [file 41467_2020_19531_MOESM1_ESM.pdf]

Supplementary Information

Artificially Innervated Self-healing Foams as Synthetic Piezo-Impedance Sensor Skins

Hongchen Guo[1,2,†], Yu Jun Tan[2,3,†], Ge Chen[2], Zifeng Wang[2], Glenys Jocelin Susanto[2], Hian Hian See[2], Zijie Yang[2], Zi Wei Lim[2], Le Yang[4], Benjamin C. K. Tee[2 3,4,5,6,*]

[1]NUS Graduate School for Integrative Sciences and Engineering (NGS), National University of Singapore, Singapore

[2]Department of Materials Science and Engineering (MSE), National University of Singapore, Singapore

[3]Institute of Innovation in Health Technology (iHealthtech), National University of Singapore, Singapore

[4] Institute of Materials Research and Engineering, Agency for Science Technology and Research, Singapore

[5] Electrical and Computer Engineering, National University of Singapore, Singapore

[6] N.1 Institute of Health, National University of Singapore, Singapore

[†]These authors contributed equally

[*] Corresponding author: benjamin.tee@nus.edu.sg

Supplementary Figures

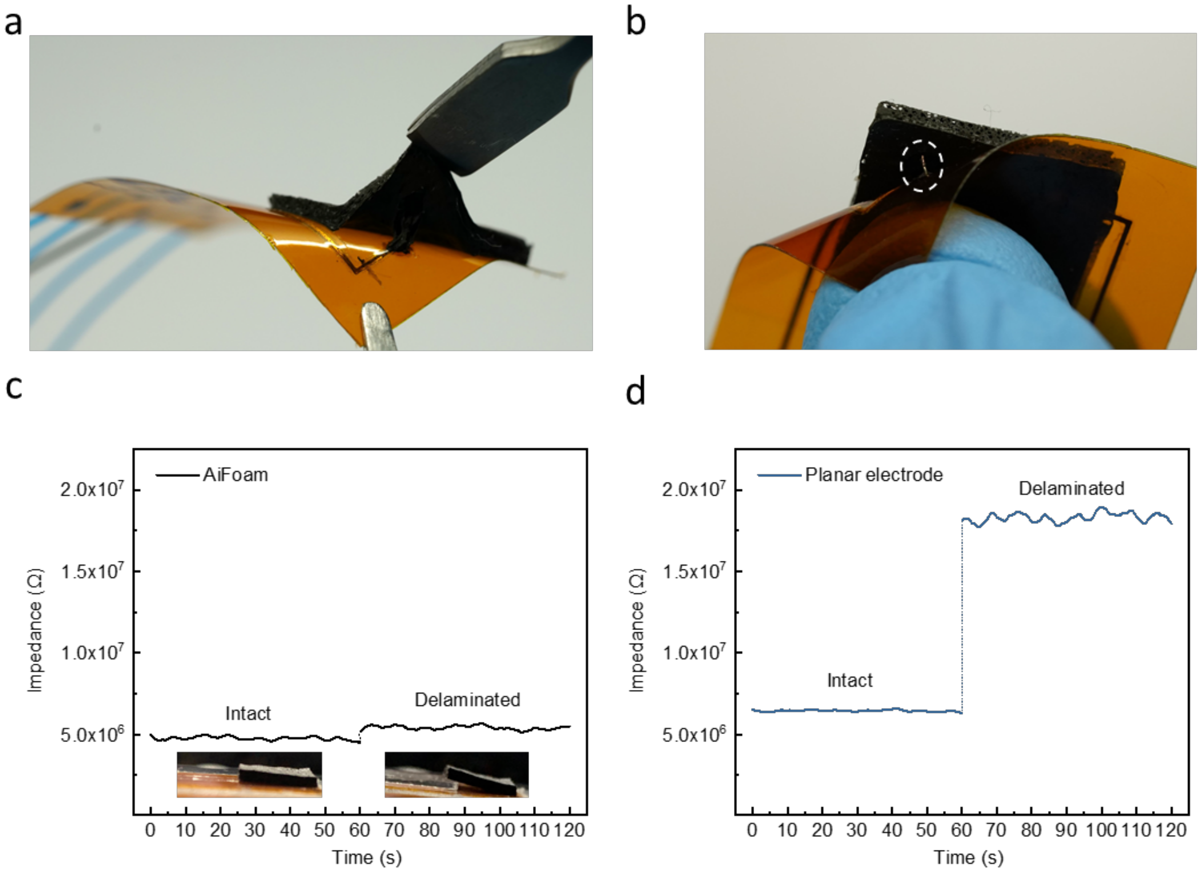

Figure 1 a, Photographs of 3D electrodes inside the AiFoam sensor. The foam material is elastic. **b,** The 3D electrodes array holds the foam materials to the electrode tightly like skewers. **c,** Graph shows that the foam material does not detach from the electrodes even if delamination occurs between the foam materials and the bottom substrate due to wear-and-tear in AiFoam. The contact between the foam and 3D electrodes is always intact. Insets: Pictures of AiFoam without delamination (left) and AiFoam delaminated from the substrate (right). **d,** This is in sharp contrast with the planar electrode sensor (Foam-2D), where the contact between the electrode and the foam could not be retained after delamination occurs.

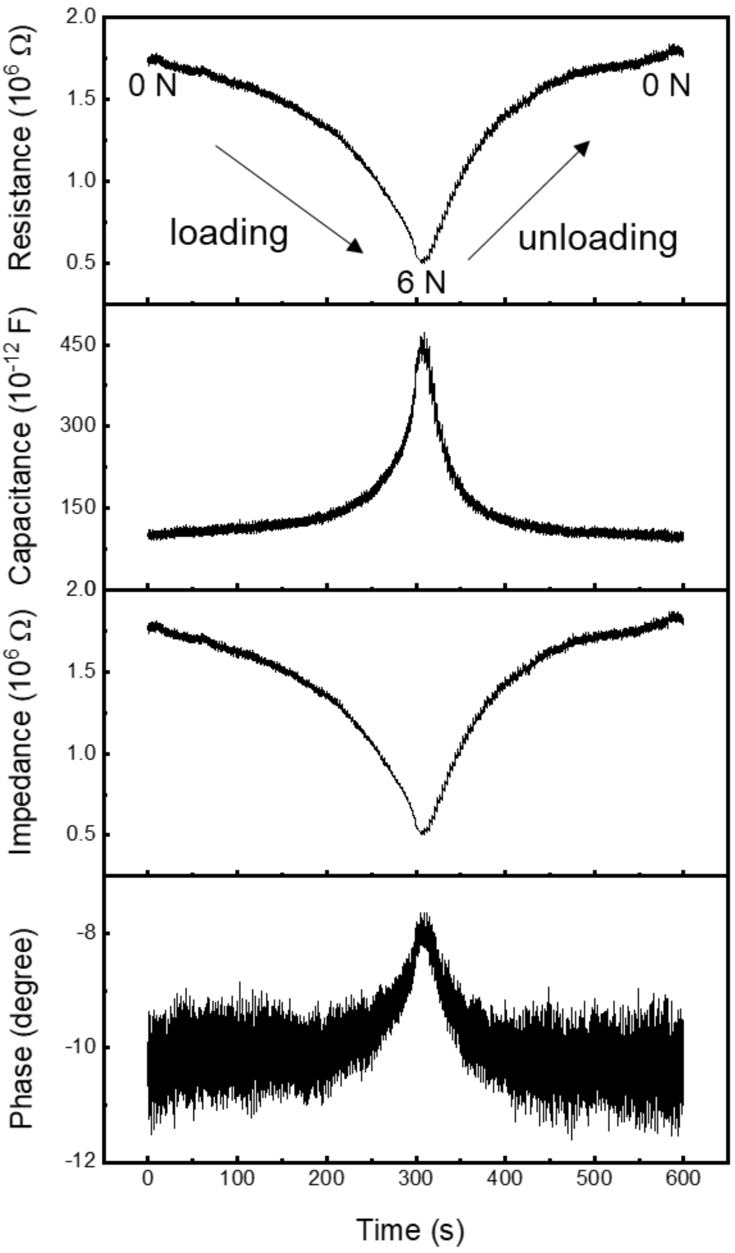

Figure 2 Piezo-impedance. Typical impedance changes of AiFoam during loading-unloading, which includes resistance, capacitance, absolute impedance and phase changes simultaneously.

Impedance is a complex number defined as:

$$Z = R + jX,$$

Where the real part R is resistance and imaginary part X is reactance. As $X = |Z| \sin \theta,$ where θ is phase angle, X is a negative value here as θ is within $0^{\circ} \sim -90^{\circ}$, indicating X here is capacitive reactance, or capacitance.

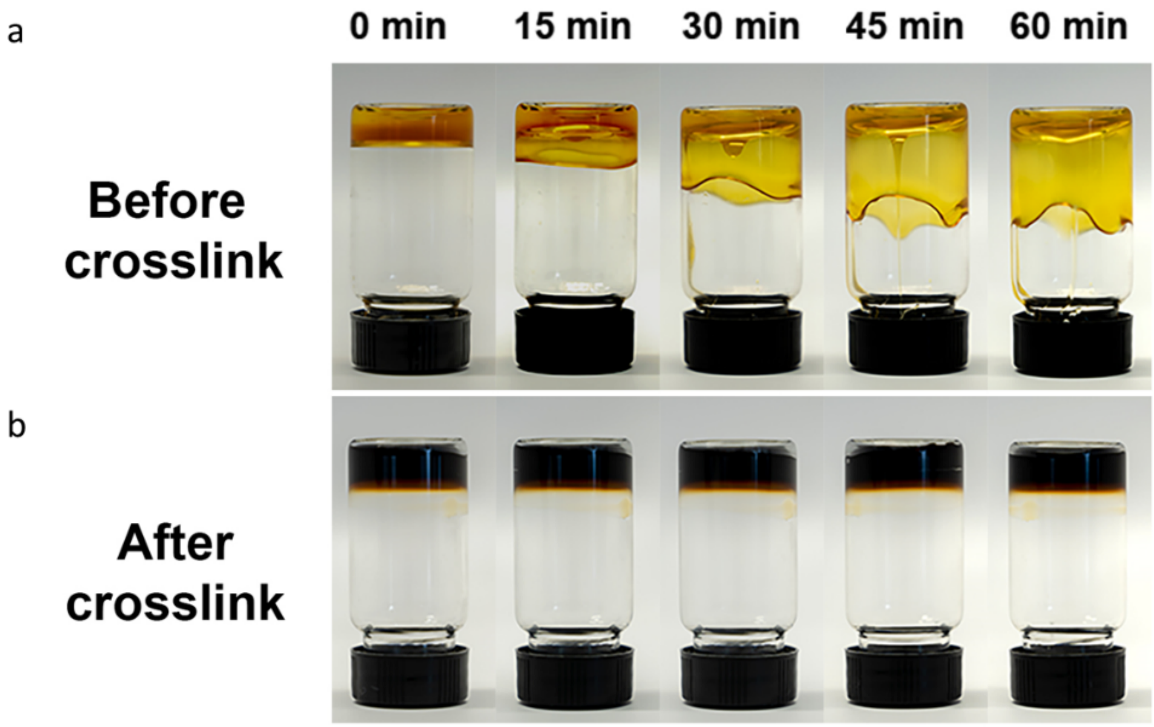

Figure 3 Photographs of **a,** PVDF-HFP-fluorosurfactant (mass ratio of PVDF-HFP: fluorosurfactant was 49: 50) and **b,** crosslinked PVDF-HFP-fluorosurfactant-DAP (mass ratio of PVDF-HFP: fluorosurfactant: DAP was 49: 50: 1), both heated at 70 ^{o}C. Fluorosurfactant is a polar solvent for PVDF-HFP. Without crosslinking, the PVDF-HFP-fluorosurfactant is flowable. After adding DAP into the mixture and crosslink at 120 ^{o}C, the mixture turned into a soft elastomer.

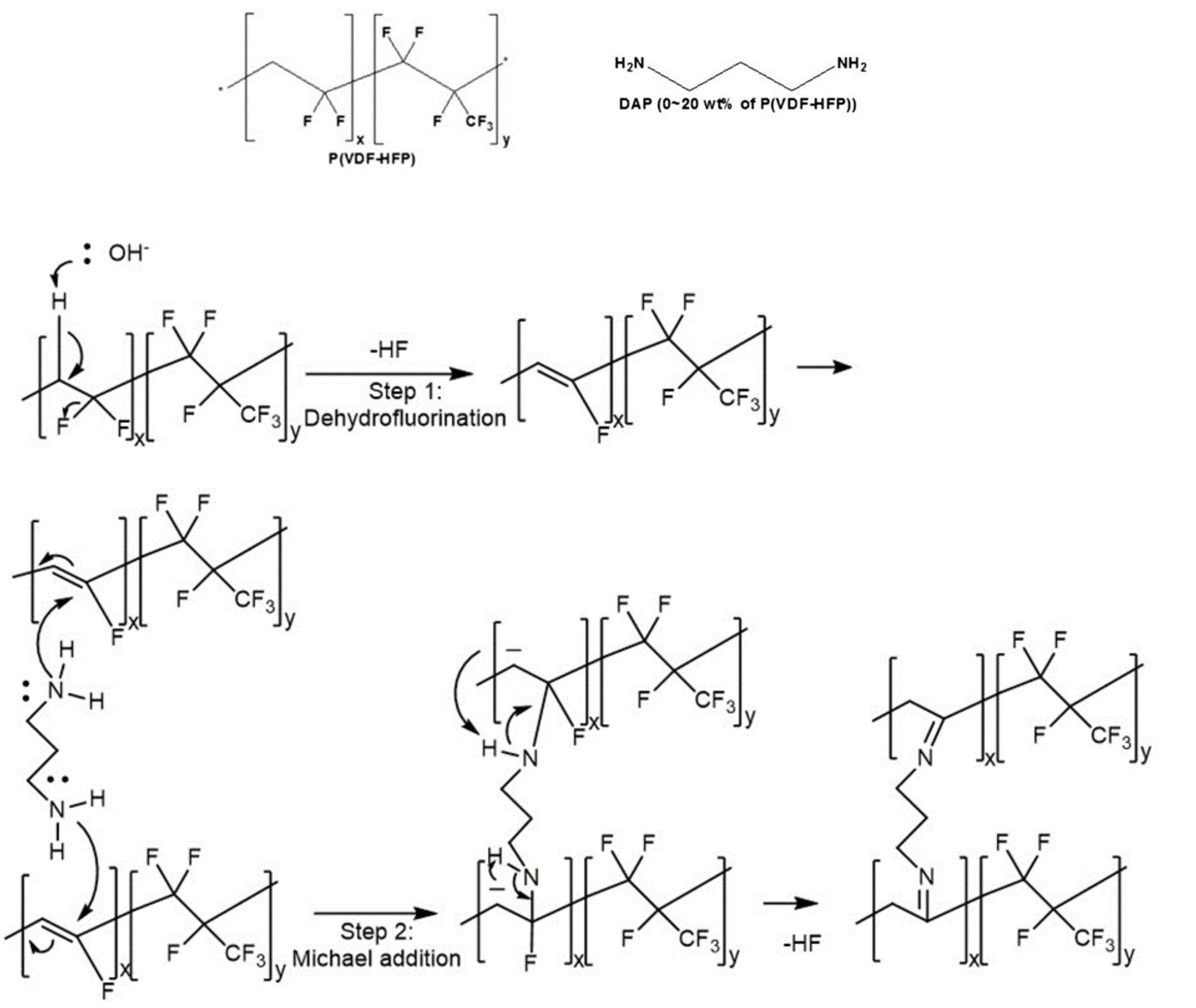

Figure 4 Crosslink mechanism. The crosslinking process contains the dehydrofluorination of PVDF-HFP and the formation of C=N bonds between the PVDF-HFP chain and DAP molecular.

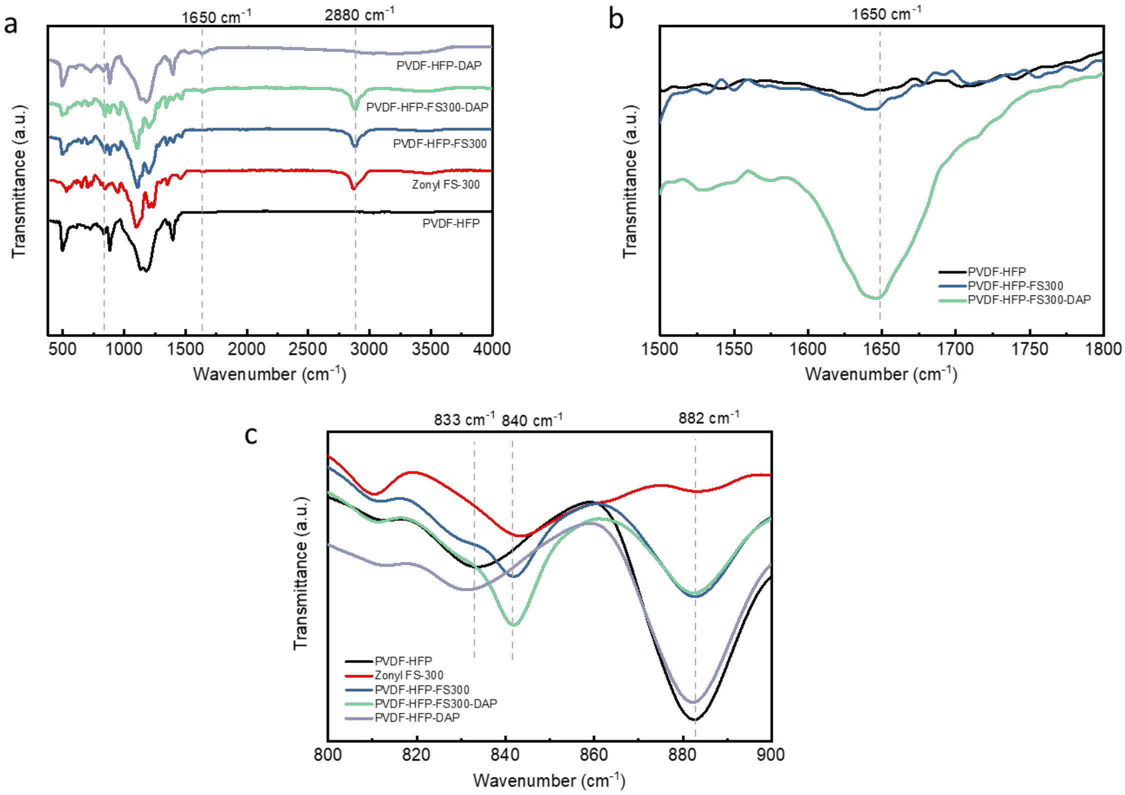

Figure 5 FTIR spectra proving the crosslinking of the polymer. **a,** FTIR spectra for PVDF-HFP, fluorosurfactant (Zonyl FS-300), PVDF-HFP-fluorosurfactant, PVDF-HFP-fluorosurfactant-DAP, and PVDF-HFP-DAP. The presence of a new peak at 2880 cm^{-1} corresponds to the CH stretching due to the presence of ether from fluorosurfactant. **b,** A new peak appeared at 1651 cm^{-1} in the FTIR spectrum of the crosslinked PVDF-HFP-fluorosurfactant-DAP polymer reflected the vibration of C=N bonds[1], proving the crosslinking by DAP. **c,** The peak located at 882 cm^{-1} indicates the amorphous phase of PVDF-HFP[2–4]. The peaks at 833 and 840 cm^{-1} corresponds to the β-phase of PVDF[2,5]. Dipole-dipole interaction between fluorosurfactant and PVDF-HFP chains can induce the rearrangement of PVDF-HFP chains, as revealed by these peaks shift in FTIR spectra.

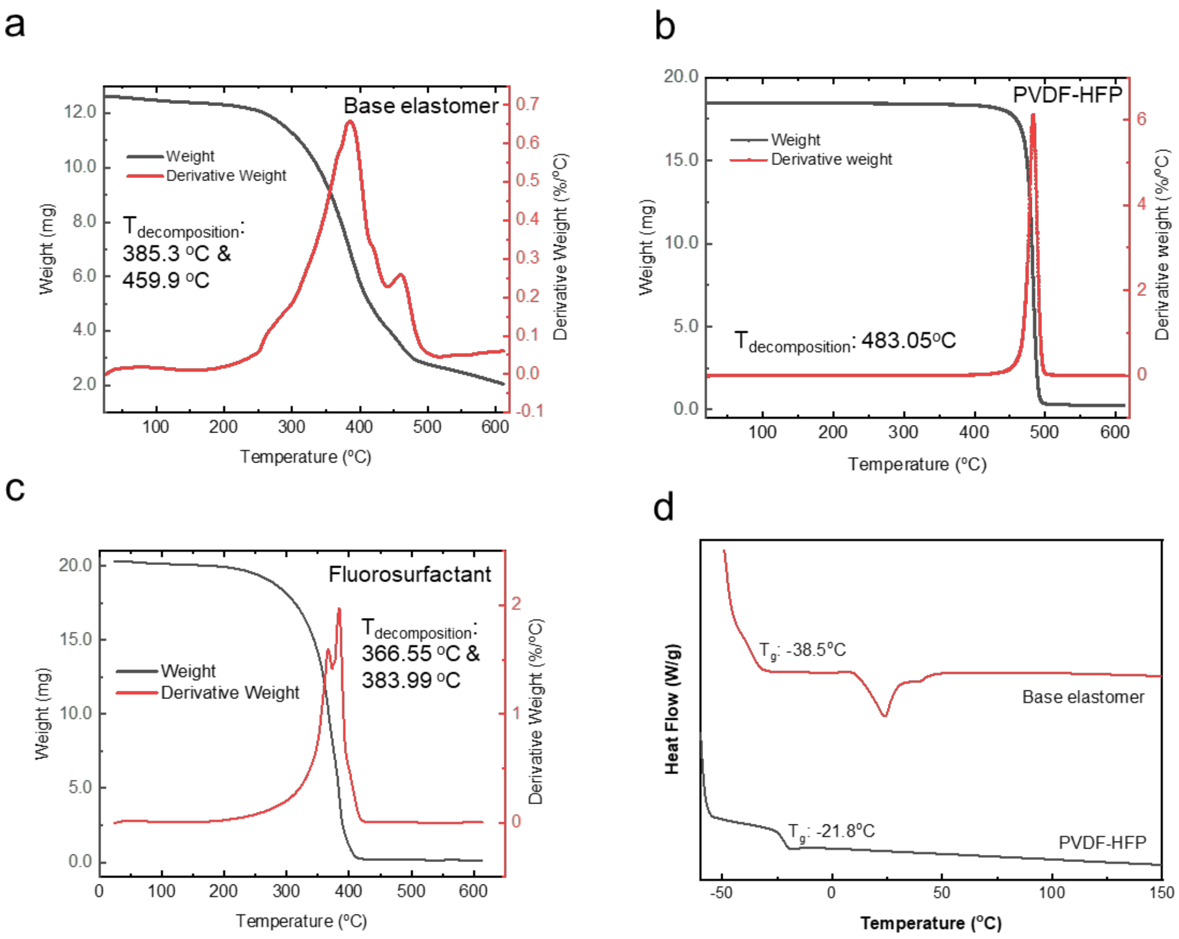

Figure 6 TGA result of **a,** PVDF-HFP-fluorosurfactant-DAP, **b,** PVDF-HFP, and **c,** fluorosurfactant Zonyl FS300. **d,** DSC spectrum of PVDF-HFP and PVDF-HFP-fluorosurfactant-DAP. Both PVDF-HFP and crosslinked PVDF-HFP-fluorosurfactant-DAP show no melting peak at ~140 °C, which is normally the melting point of crystalized PVDF-HFP, indicating the materials are amorphous. A shift of glass transition temperature (T_g) from -21.8 °C of PVDF-HFP to -38.5 °C of crosslinked PVDF-HFP-fluorosurfactant-DAP was observed, confirming the plasticizer function of fluorosurfactant.

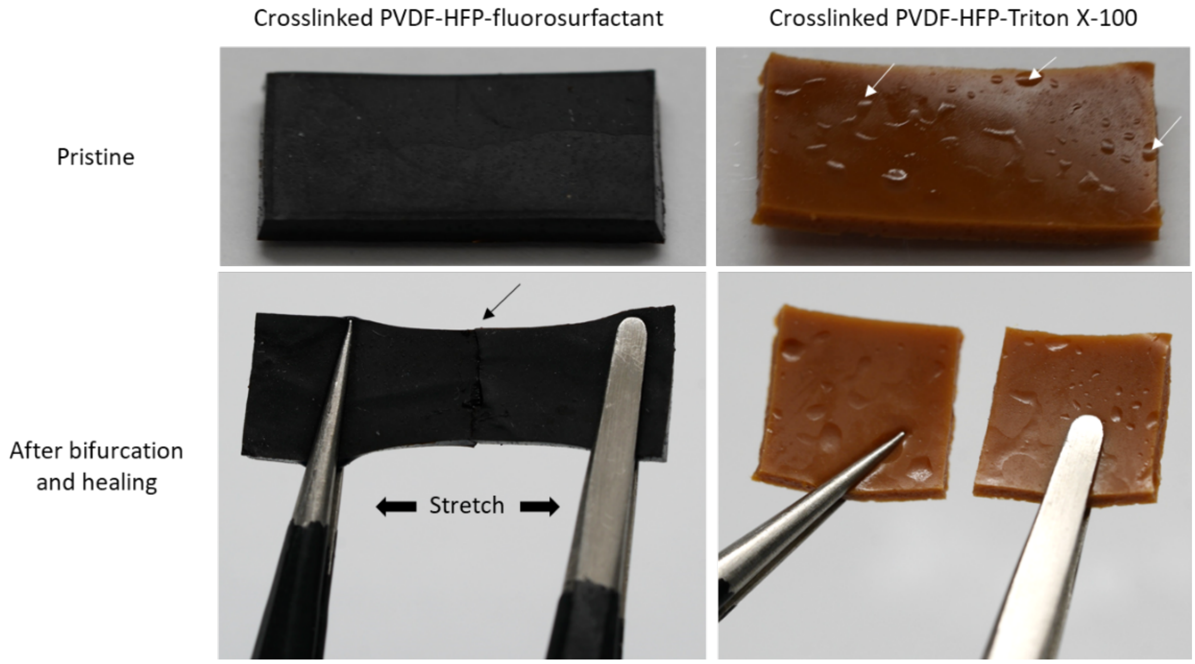

Figure 7 Photographs of self-healing performance of **left:** PVDF-HFP-fluorosurfactant-DAP and **right:** PVDF-HFP-hydrocarbon surfactant-DAP. The hydrocarbon surfactant (Triton X-100) did not blend well with the fluoropolymer, leaching out from the polymer as shown by the white arrows. PVDF-HFP-hydrocarbon surfactant-DAP did not self-heal from bifurcations.

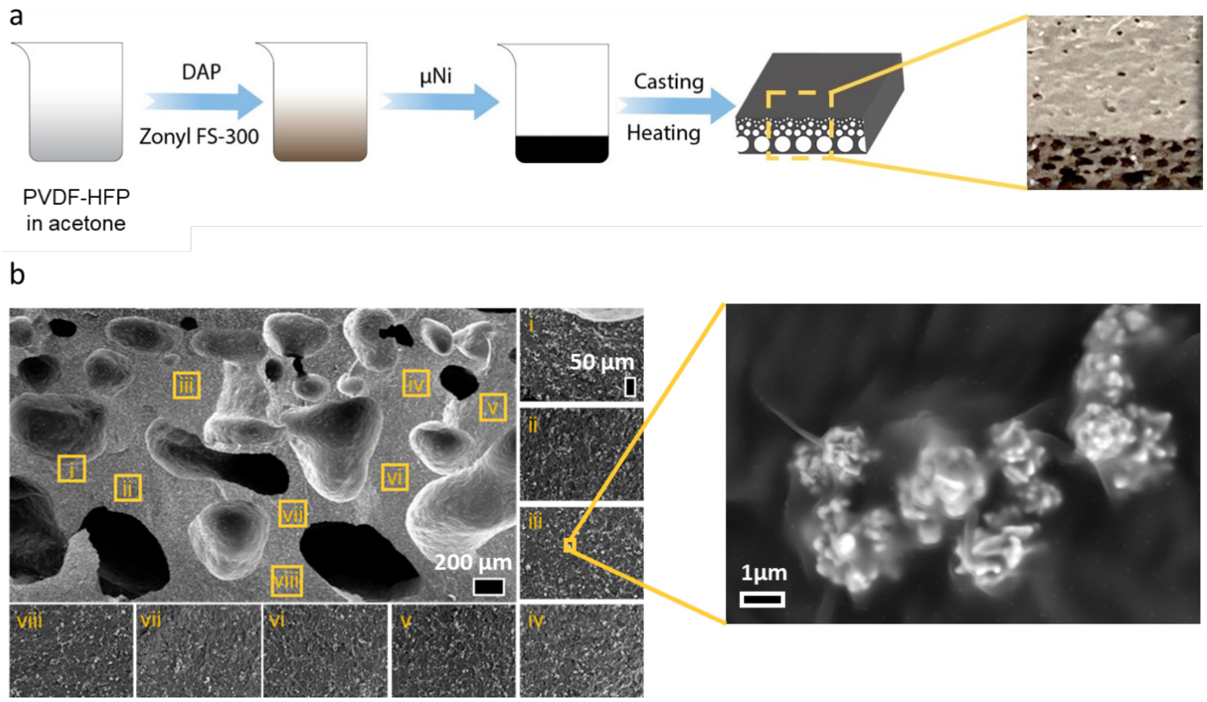

Fig 8 a, Fabrication process of the resistive foam material. PVDF-HFP was dissolved in acetone and mixed with Zonyl FS-300 and DAP in sequence. After μNi was added, the mixture was directly cast into a glass mold, then immediately heated at 70 °C. When heated at 70 °C, acetone solvent evaporated, leaving pores inside the polymer. After increasing the temperature to 120 °C, the crosslinking process of PVDF-HFP chains by DAP was accelerated and completed. **b,** SEM images of Foam 25 μNi. Zoomed-in images of foam material reveal the uniformity and consistency of μNi particles dispersing inside the elastomer matrix.

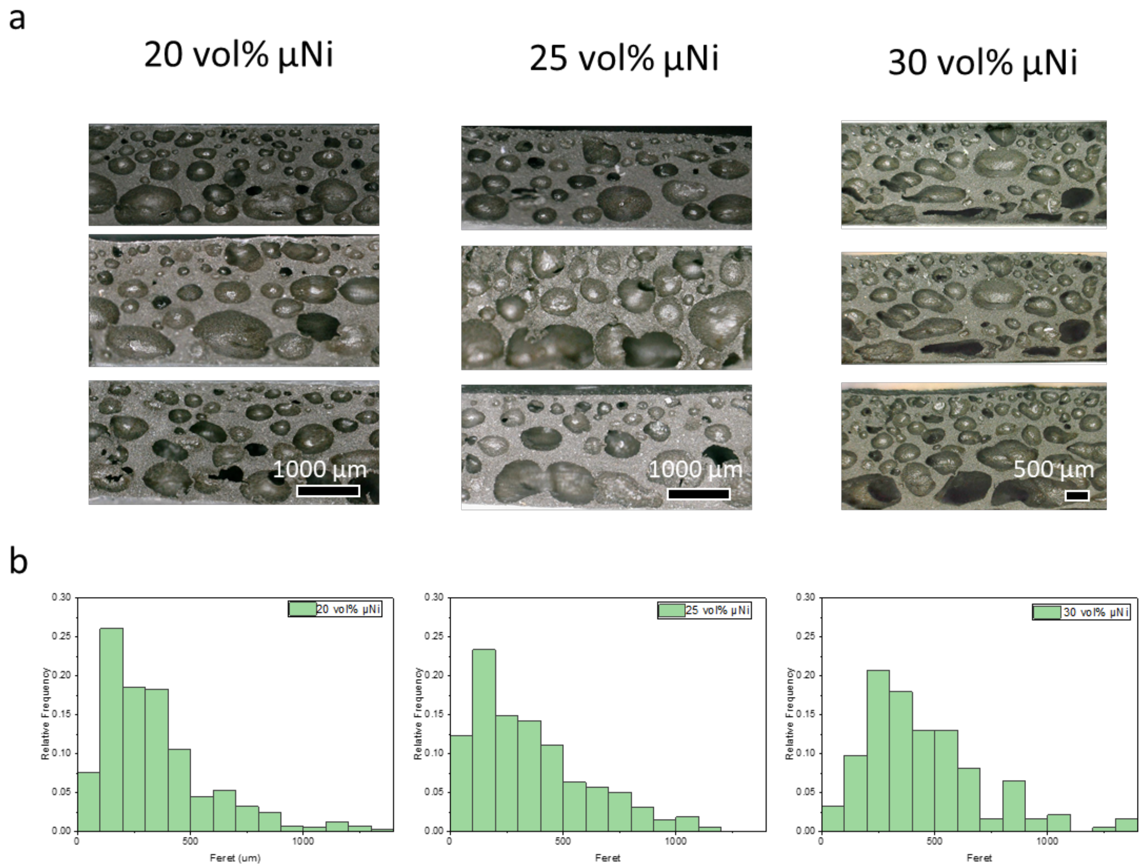

Figure 9 a, Optical microscope images of **Left:** Foam 20 µNi. **Middle:** Foam 25 µNi. **Right:** Foam 30 µNi. The images depict the pore structure in AiFoam as bigger voids at the bottom and smaller on top. All 3 samples in each group of µNi loading show the same structure, revealing the fabrication process is repeatable. **b,** Pore size distributions of the above images.

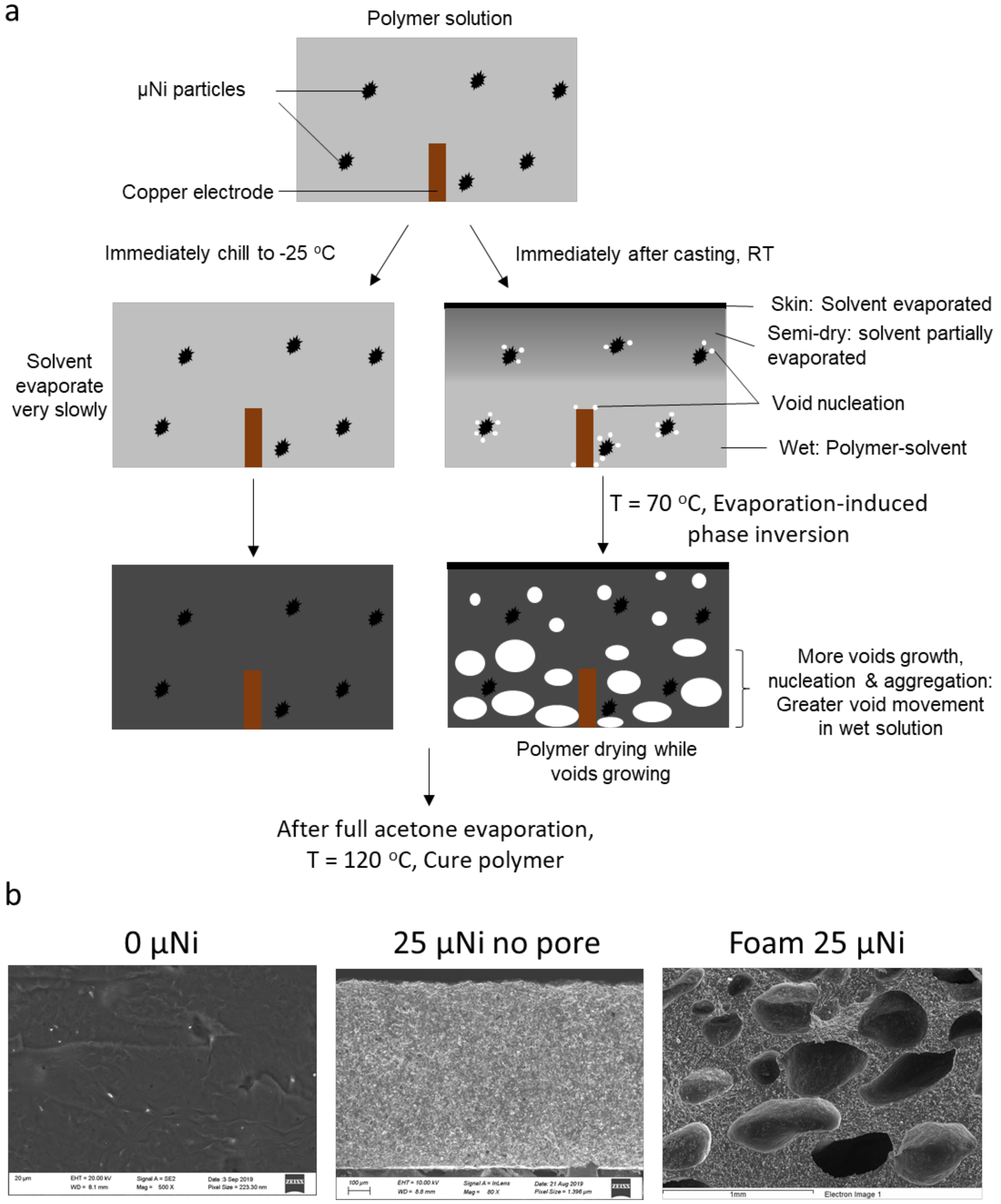

Figure 10. Mechanism of the voids' formation in the self-foaming AiFoam materials. **a,** Voids nucleate at the µNi particles/polymer solution and electrode/polymer solution interfaces when the solvent evaporates. The voids grow and aggregate as the solvent evaporates at 70 °C due to the evaporation-induced phase inversion. Voids at the top stop growing subsequently because the solvent evaporates more easily due to the air exposing surface. Voids at the bottom keep nucleating and growing until all the solvent escape from the polymer. Meanwhile, to make µNi samples with no pores, we formed the polymer at low temperatures for slow solvent evaporation. **b,** SEM images of **Left:** base elastomer without µNi, **Middle:** 25 µNi with no pores, and **Right:** Foam 25 µNi.

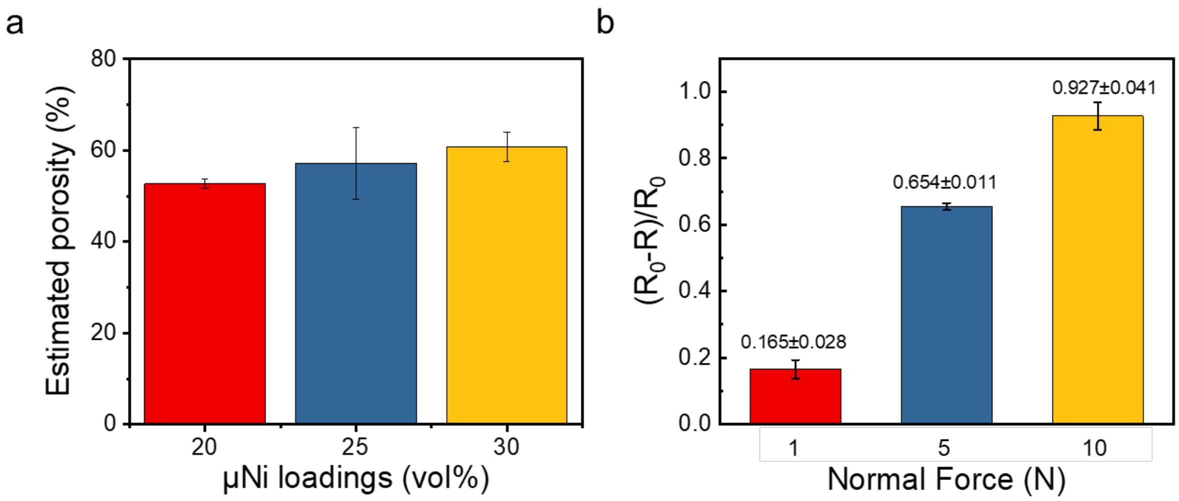

Figure 11 a, Porosity of AiFoam with different µNi loadings and **b**, consistency of sensor performance across 3 different batches. Resistance change of AiFoam 30μNi samples from 3 different batches (under normal force of 1 N, 5 N and 10 N) revealed good consistency of sensor performance across batches.

From Supplementary Figure 8 and Figure 9 we can see all samples have bigger pores at the bottom and smaller pores on top. This can be related to the void formation theory (see Supplementary Figure 10). Meanwhile, all the pore size distributions calculated by ImageJ show similar trends. For samples with 20 vol% µNi (20Ni) and 25 vol% µNi (25Ni), the most distributed pores are within the range of 100-500 µm. The range is extended to 100-700 µm for the samples with 30 vol% µNi (30Ni), indicating pore size increase with more µNi. Nevertheless, comparing with 20Ni and 25Ni samples, the 30Ni sample shows a more irregular pore shape, especially for pores with large size. This is because more void nucleation sites are provided with more µNi, these voids can grow bigger and merge when the polymer solution was heated. As the voids growing, the polymer was solidifying slowly and confining the size and shape of the voids. Voids with different sizes and shapes merge and lead to irregular bigger shape pores at the bottom. Moreover, more µNi loading can bring more void nucleation sites and lead to porosity increase.

Porosity was calculated based on the area ratio of pores to the materials based on the cross-section of images in Figure S8a. With more µNi loading, the porosity increased from 52.7% (20Ni) to 60.8% (30Ni).

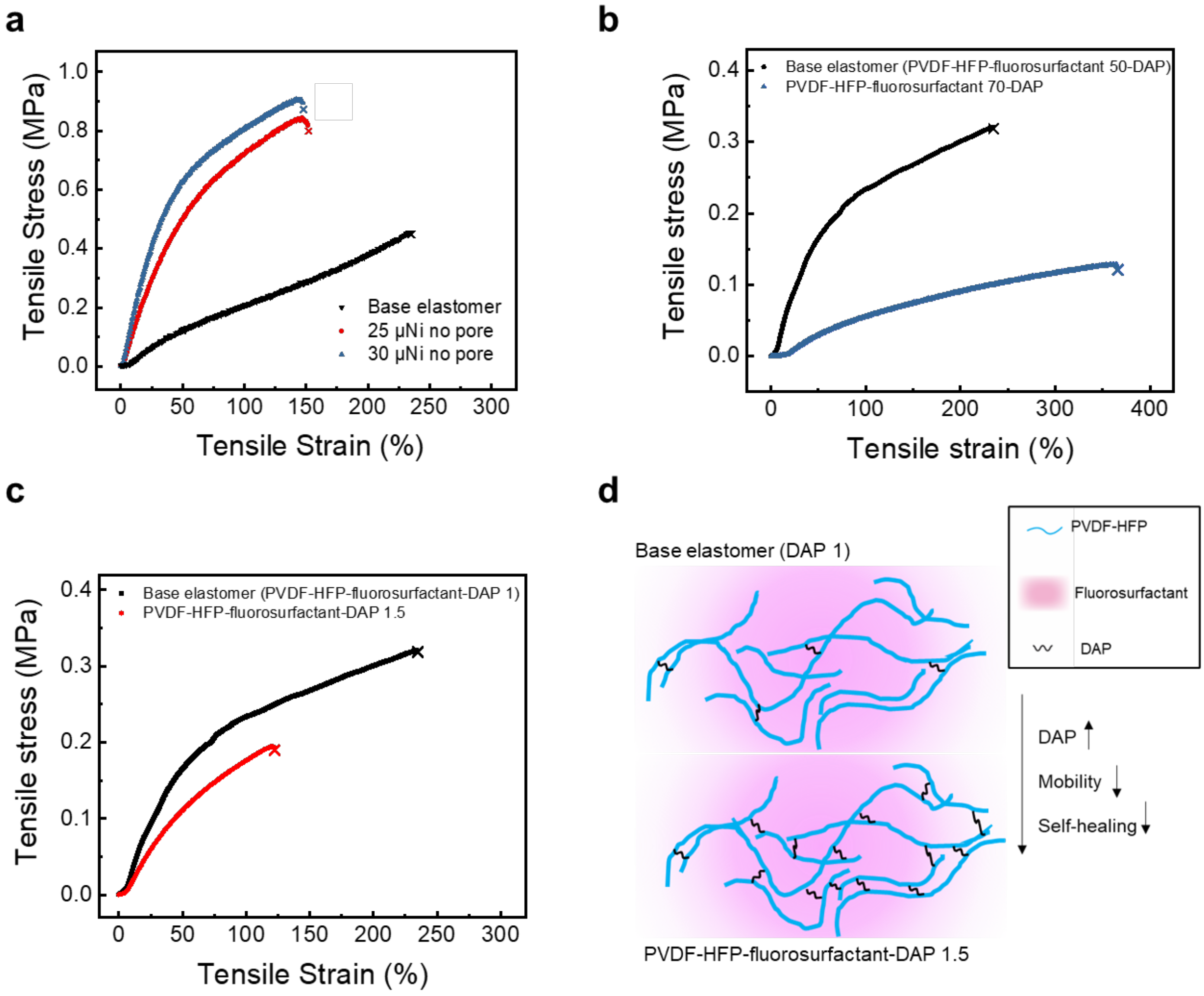

Figure 12 Tensile test results of **a,** no pore samples with varying μNi loadings in base elastomer, **b,** base elastomer samples with varying fluorosurfactant loadings (50 vs 70 wt%), **c,** base elastomer samples with varying DAP loadings (1.0 vs 1.5 wt%), **d,** schematics to explain the effects of DAP on the self-healing ability of polymer. As shown in Figure S12a, the material became stiffer with more μNi loading. Figure S12b shows that with the increasing fluorosurfactant loading from 50 wt% to 70 wt%, a huge decrease in modulus was observed. The material became too soft to be handled. With 30 wt% of fluorosurfactant, the self-healing ability of the material deteriorated. With the increase in DAP loading from 1 wt% to 1.5 wt% as shown in Figure 12c, the maximum elongation of the material decreased significantly attributed to the increment in the crosslinking of the polymer. The excessive crosslink also decreases the mobility of the polymer, hence reduces its self-healing ability. If the DAP loading was reduced to 0.5 wt%, the material flows and is not free-standing.

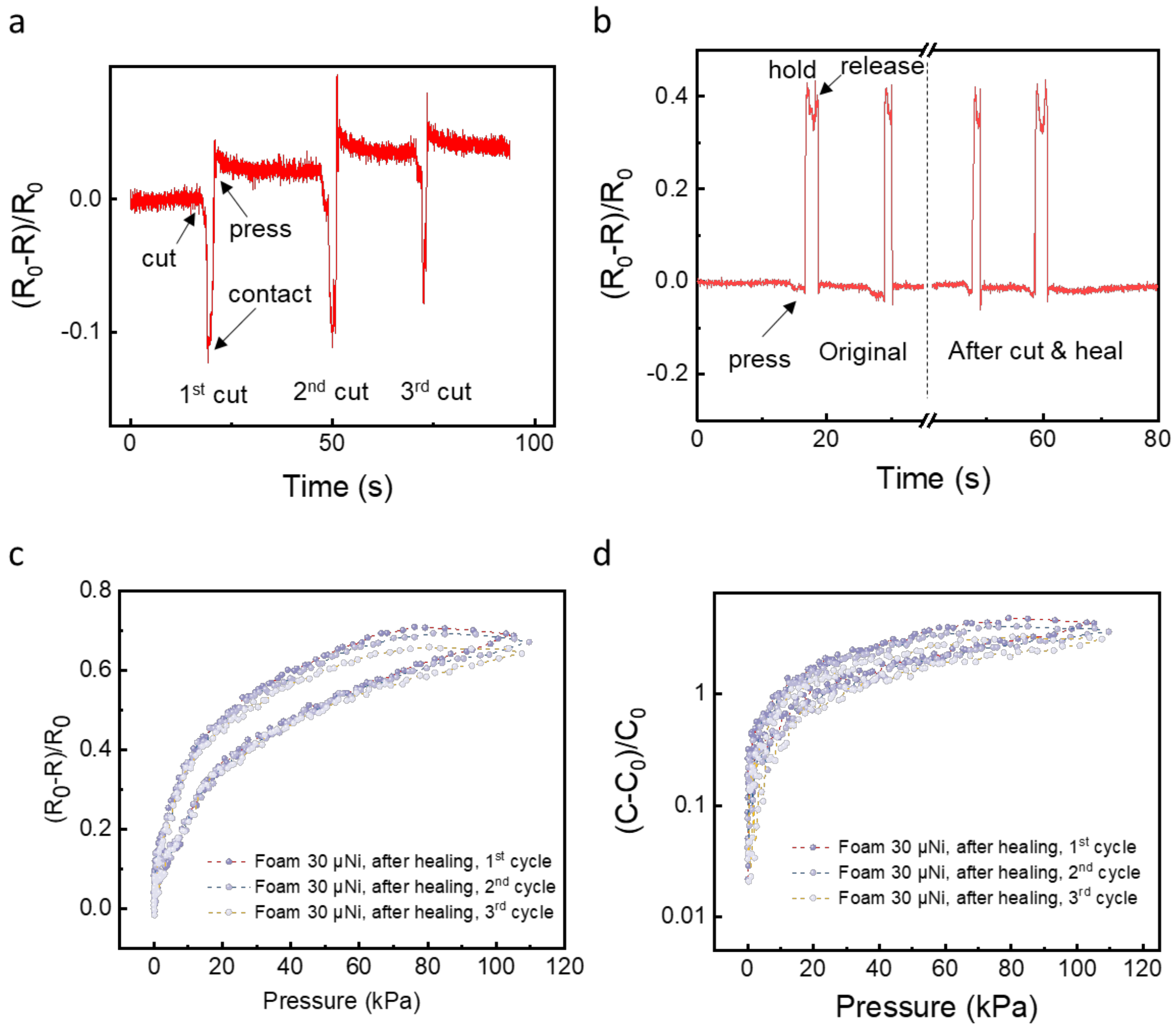

Figure 13 a, Self-healing of the resistance of Foam 25 µNi upon bifurcation and contact. **b,** Self-healing of the pressure sensing of Foam 25 µNi after bifurcation and self-heal. **c,** Resistance change vs normal pressure on Foam 30 µNi after self-healing. **d,** Capacitance change vs normal pressure on Foam 30 µNi after self-healing.

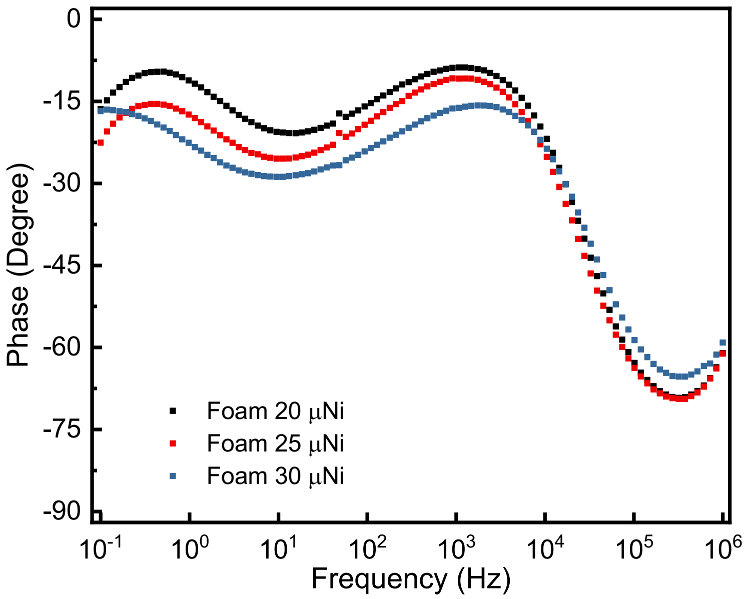

Figure 14 Bode plots of AiFoam materials with different μNi loadings. Two peaks in the range of 10^0 to 10^2 Hz and 10^5 to 10^6 Hz, respectively, are consistent with the two semicircles shown in the Nyquist plots. The Bode plots of the samples with different μNi have similar shape and position of the peaks, although minor shifts in the phase angles were observed. This indicates that the samples with different μNi loading may be modelled and explained using the same equivalent circuit but with different parameters. The graphs reveal the ion-conductive-like behaviour of foam material.

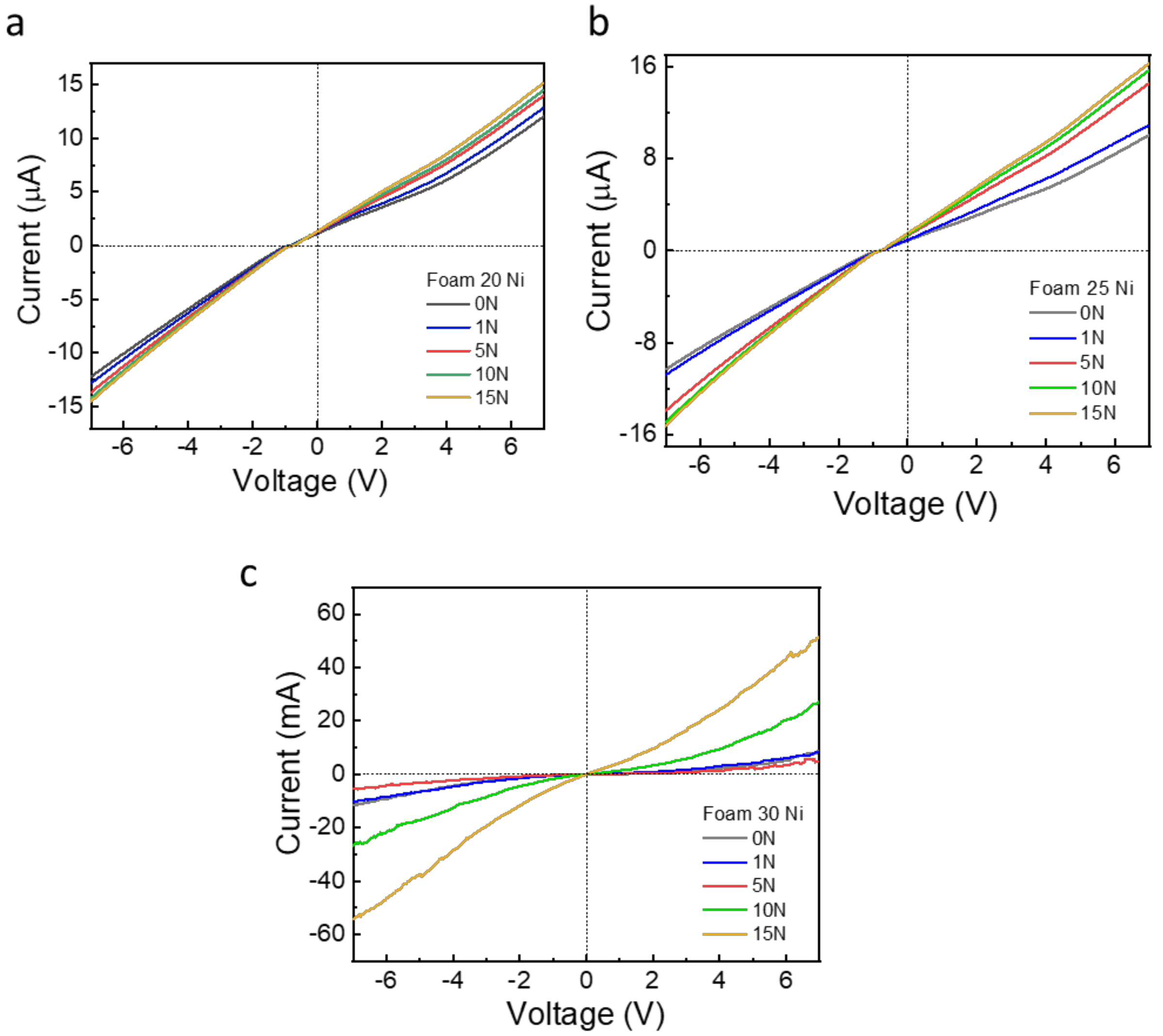

Figure 15 I-V curves of **a,** Foam 20 µNi, **b,** Foam 25 µNi, and **c,** Foam 30 µNi under different loadings (F = 0-15 N). Graphs indicate the electron-conductivity of the material, which can be related to quantum tunnelling. For example, Foam 25 Ni shows the non-linear I-V behaviour and the slope increased with increasing the loading force, which means the resistance of the material decrease with the increasing pressure. All the samples show similar behaviour. This atypical non-linear I-V phenomenon indicates that the conductivity mechanism of this foam is not pure Ohmic conduction (with linear I-V correlation). The non-linearity may also due to the additional contribution of the quantum tunnelling in the materials[6].

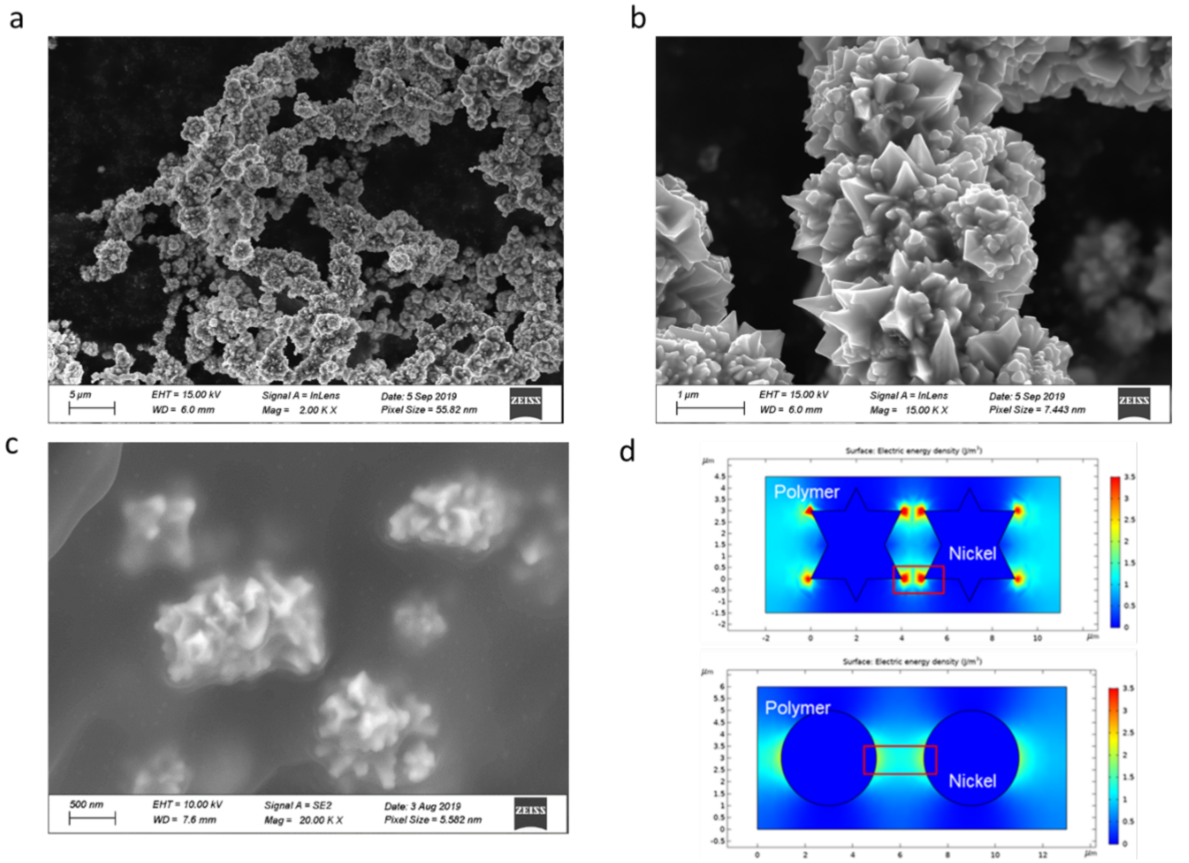

Figure 16 SEM image of **a,** μNi particles and **b,** zoom-in view of μNi particles. **c,** SEM image of μNi particles buried in the foam polymer. **d,** COMSOL simulations urchin-like μNi particles, and spherical μNi particles. Urchin-like nickel microparticles with nano-spikes on the particle surface were evenly dispersed in the polymer matrix. Compared with smooth spherical nickel particles, these nanostructures would enhance the local electric field resulting in higher conductivity. The surface charge density had an obvious positive correlation with the curvature of the metal surface as verified by the COMSOL simulation. The electric energy density is much higher between the sharp points of particles.

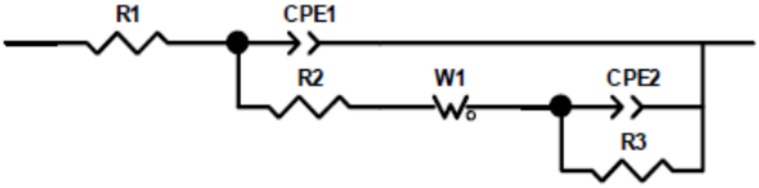

Figure 17. The equivalent circuit for the PVDF-HFP-fluorosurfactant-DAP-μNi materials. The R_1 represents bulk resistance, while CPE_1 and R_2 represent the double-layered capacitance and the polarization resistance of one of the constituent materials, respectively. The CPE_2 and R_3 represent the other double-layered capacitance and its polarization resistance of the other constituent material, whereas the W_1 element indicates the mass transport process inside the material. It is believed that the mass transport process correlates closely to the surfactant, which acts as both the solvent to PVDF-HFP polymer and the small molecules with charge asymmetry. Consequently, we hypothesize that the surfactant molecules can either migrate/diffuse or assist the PVDF-HFP chains to migrate/diffuse under either an electric field or concentration gradient.

Parameters of the equivalent circuits are shown in Supplementary Table 3-4. It can be found that the values of R_1 show a clear correlation with Ni particle concentrations. In particular, the samples without pores showed much lower R_1 values than those with pores, as shown in Supplementary Table 3 and 4. It can be also observed that the values of R_1 for composites of 30 and 35 vol% μNi loading fractions without pores are indeed quite close, probably due to the percolation of the conductive fillers, which is different with the samples contains pores with the same μNi loading fractions. Besides, in both cases, either containing pores or not, the values of capacitance from CPE_1 are much smaller than CPE_2, indicating a different contribution of the capacitance from the constituent materials. Given that the above-mentioned discussion is correct, the CPE_2 can be attributed to the capacitance contributed by surfactant transport, whereas the CPE_1 may originate from PVDF-HFP polymer. The values of the CPEs also show consistency with the μNi particles and surfactant concentrations, respectively. The values of CPE_1 increase with μNi particle concentration from 3.39 x 10^{-11} to 1.85 x 10^{-10} and 1.08 x 10^{-10} to 4.07 x 10^{-10}, respectively for samples with or without pores, when μNi particle concentration increase from 20 to 35 vol%. This may be ascribed to the increased surface area between μNi particles and PVDF-HFP. On the other hand, the values of CPE_2 increase from 6.82 x 10^{-8} to 1.52 x 10^{-8} with surfactant concentrations increase from 40 to 70 wt%, whereas the one without any μNi addition gave rise to a large CPE_2 of 4.67 x 10^{-6}.

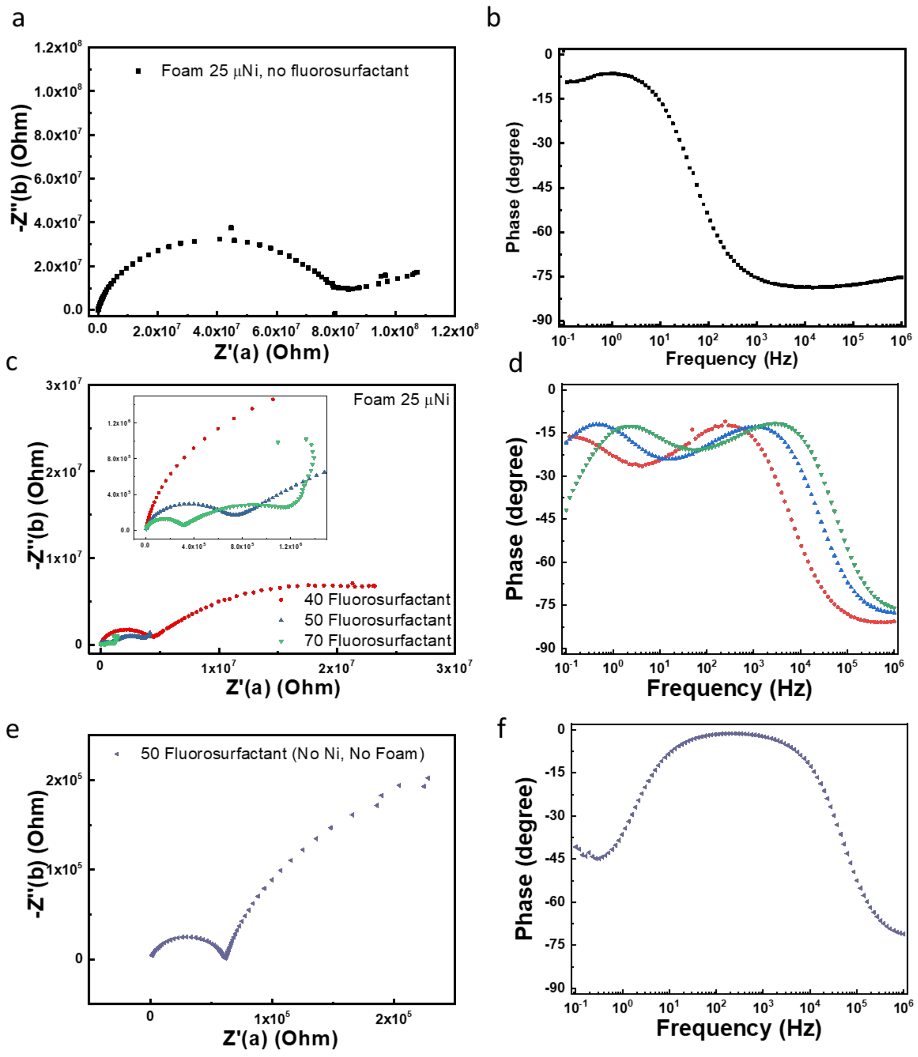

Figure 18 a, Nyquist plot and **b,** Bode plot of Foam 25 μNi with no addition of fluorosurfactant. The plots show different electrical behaviour as compared to our Foam 25 μNi with fluorosurfactant addition. Only one obvious semicircle can be seen within the range of the frequency tested in the Nyquist plot. **c,** Nyquist plot and **d,** Bode plots for samples with different fluorosurfactant concentrations. **e,** Nyquist plot and **f,** Bode plot for the sample without μNi. Samples with various fluorosurfactant concentrations significantly affect the impedance of the composite materials. The radii of the first semicircles that appear in the leftmost of the Nyquist plot decreased dramatically from around 4.5 MOhm to less than 500 kOhm when the surfactant concentration increased from 40 to 70 wt%, respectively, whereas the radii of the second observable semicircles also decreased accordingly. On the other hand, Figure 18d shows obvious right-shift of the peaks, from around 10^1 to 10^2 Hz, with an increasing amount of fluorosurfactant added. This indicates that the electrical performance of the composites may correlate closely to the surfactant. The sample without μNi particle incorporation shows two peaks at low and high frequencies range, respectively, indicating that there are two separate capacitive mechanisms.

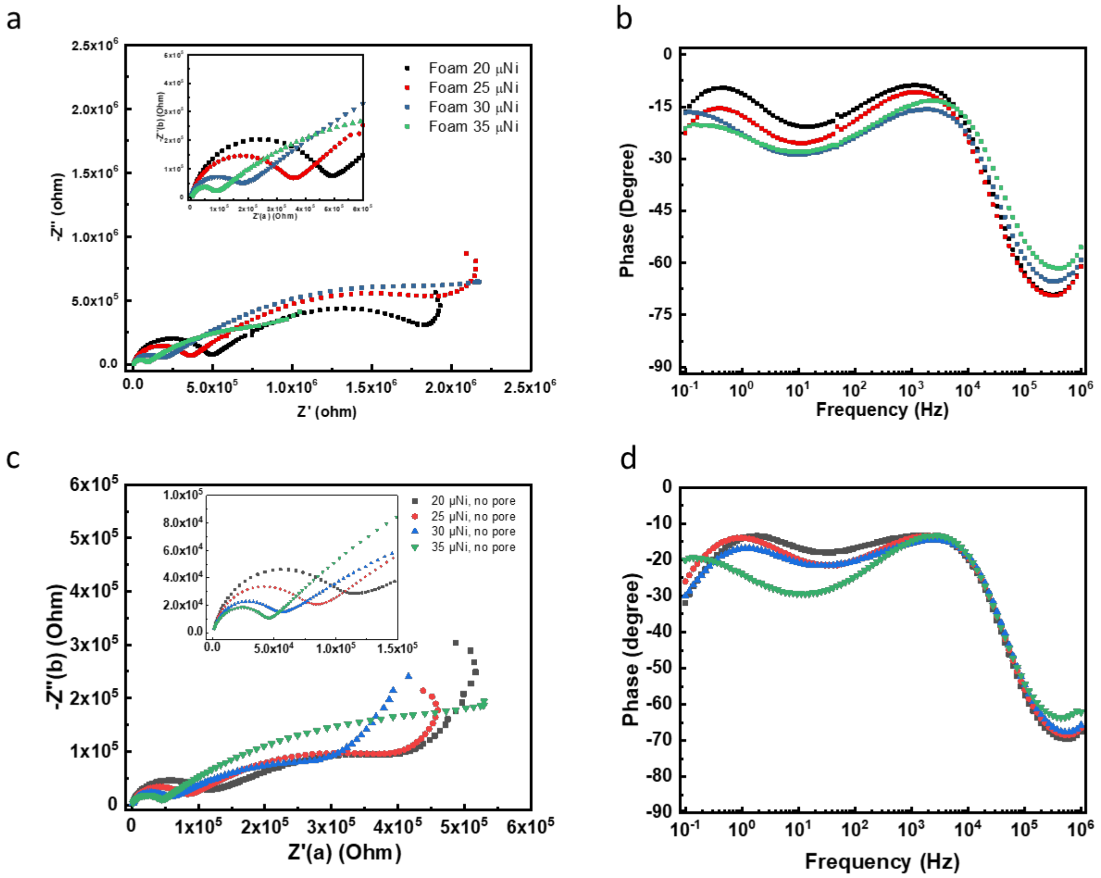

Figure 19 Nyquist and Bode phase plots of foam and non-porous materials of different μNi loadings. **a,** Nyquist plots and **b,** Bode plots for foam samples with different μNi. **c,** Nyquist plot, and **d,** Bode plots for nonfoam samples different μNi. To test the impedance of the materials, samples with different Ni concentrations were cut into 1×1 cm^2 size and sandwiched by two gold sheets, which were then connected to the MFIA LCR meter for impedance scanning from 10^{-1} to 10^6 Hz.

The samples without pores exhibited much smaller radii on the semicircles at the high-frequency range, which decreased from 120 kOhm to 50 kOhm when μNi loading increased from 20 to 35 vol%, respectively. This is consistent with the fact that the materials become denser without the pores, which is favorable for the electrical conduction within the materials. The Bode phase plots of composites without pores show that the two peaks with positions similar to those samples without pores remain in the curves. The shape of the curves and the positions of the peaks show high similarity when comparing with the samples with pores. The difference in the sample with 35 vol% μNi loading may arise from the percolation of μNi particles. This indicates that although there might be some minor variations, such as a slight shift in the phase angles and positions of the peaks, the dominating mechanism may remain invariant when pores are removed from the sample.

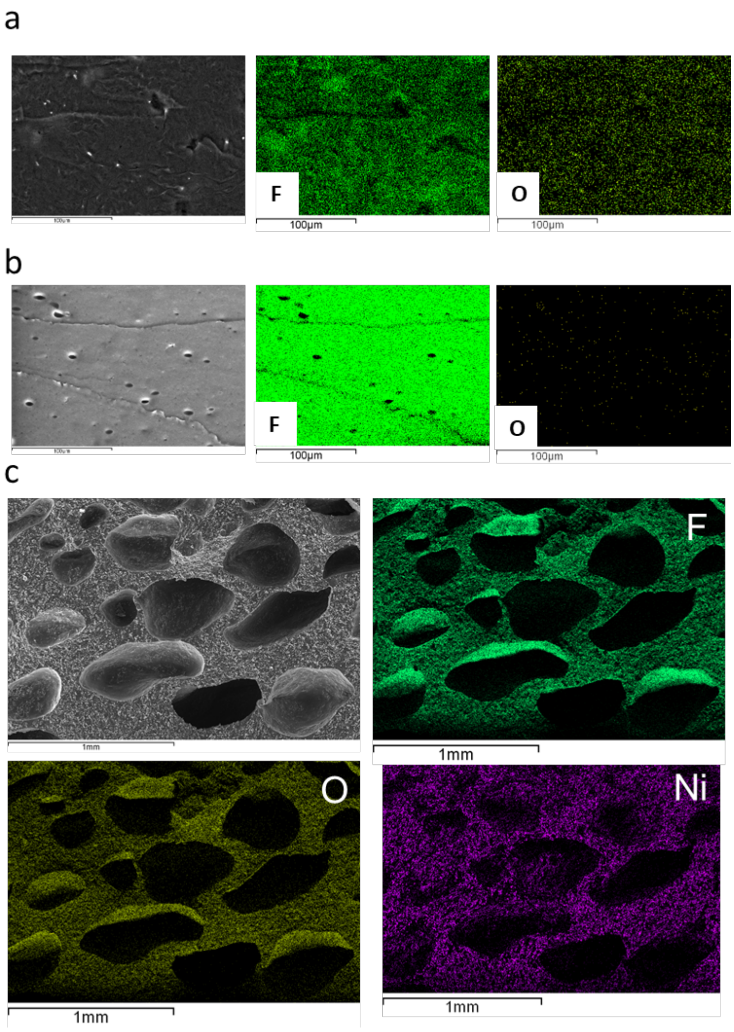

Figure 20 EDS mapping of **a,** base elastomer, **b,** base elastomer without fluorosurfactant, and **c,** Foam 25 μNi. Oxygen concentration was negligible when no fluorosurfactant was added, revealing the distribution of fluorosurfactant in Foam material. μNi particles are dispersed throughout the foam material.

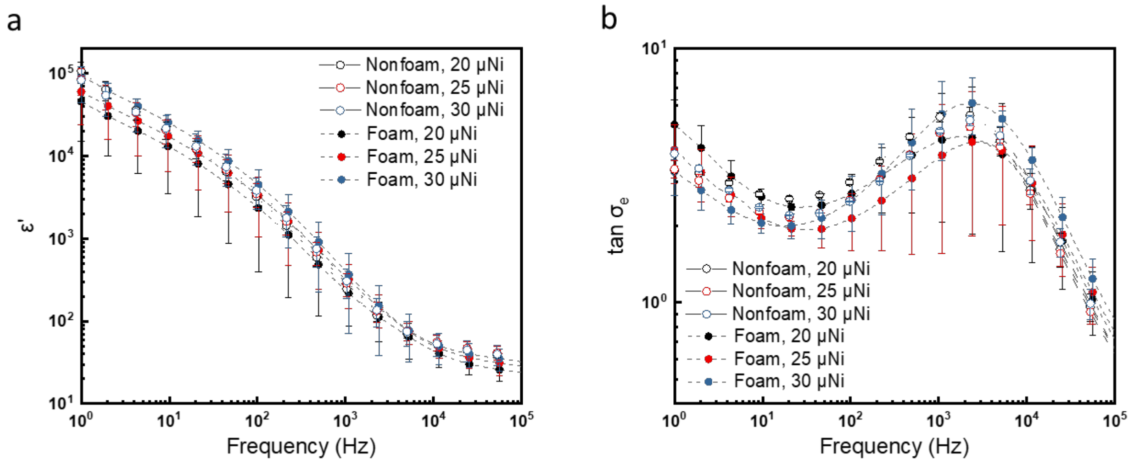

Figure 21 a, Permittivity and **b,** loss tangent of materials with different μNi concentration. The error bars are calculated based on three samples.

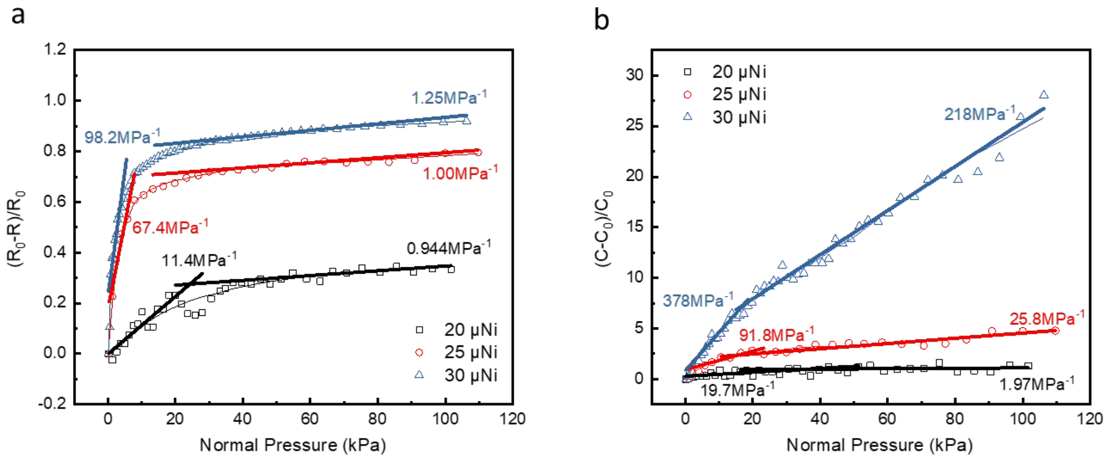

Figure 22 Sensitivity of AiFoam. a, Resistance change vs normal pressure. **b,** capacitance change vs normal pressure. The sensitivity of resistance change and capacitance change was calculated as shown below:

$$S_R = \frac{\delta\left(\frac{(R_0 - R)}{R_0}\right)}{\delta P}$$

$$S_C = \frac{\delta\left(\frac{(C - C_0)}{C_0}\right)}{\delta P}$$

Both the S_R and S_c increased with the increasing µNi loadings. When P < 10 kPa, the S_R reached 98.2 MPa^{-1} for AiFoam 30 µNi, which is ~9 times of the sensitivities (11.4 MPa^{-1}) of the AiFoam 20 µNi. Meanwhile, the S_c of AiFoam 30 µNi (378 MPa^{-1}) increased even higher, at ~19 times compared to the AiFoam 20 µNi (19.7 MPa^{-1}). The change of S_R with different µNi loadings became gentle when 10 kPa < P <100 kPa, increasing from 0.944 MPa^{-1} (AiFoam 20 µNi) to 1.25 MPa^{-1} (AiFoam 30 µNi). Nevertheless, the change in S_c was still high at the same pressure range, increasing from 1.97 MPa^{-1} (AiFoam 20 µNi) to 218 MPa^{-1} (AiFoam 30 µNi). The S_c is always higher than S_R, which can be attributed to the incorporation of fluorosurfactant and µNi. The resistive behaviour was built on the percolation of µNi in the base elastomer. When P < 10 kPa, the porous structure in our foam was compressed first and caused a big change in resistance. When the materials were compressed further, the resistance change slowed down and led to a small S_R change. Meanwhile, The capacitive behaviour was contributed by the interactions and interfaces of the electrode/surfactant, μNi/surfactant, and µNi/PVDF-HFP. More µNi loadings can bring more µNi/surfactant and µNi/PVDF-HFP interfaces, leading to a high S_c even when P > 10 kPa.

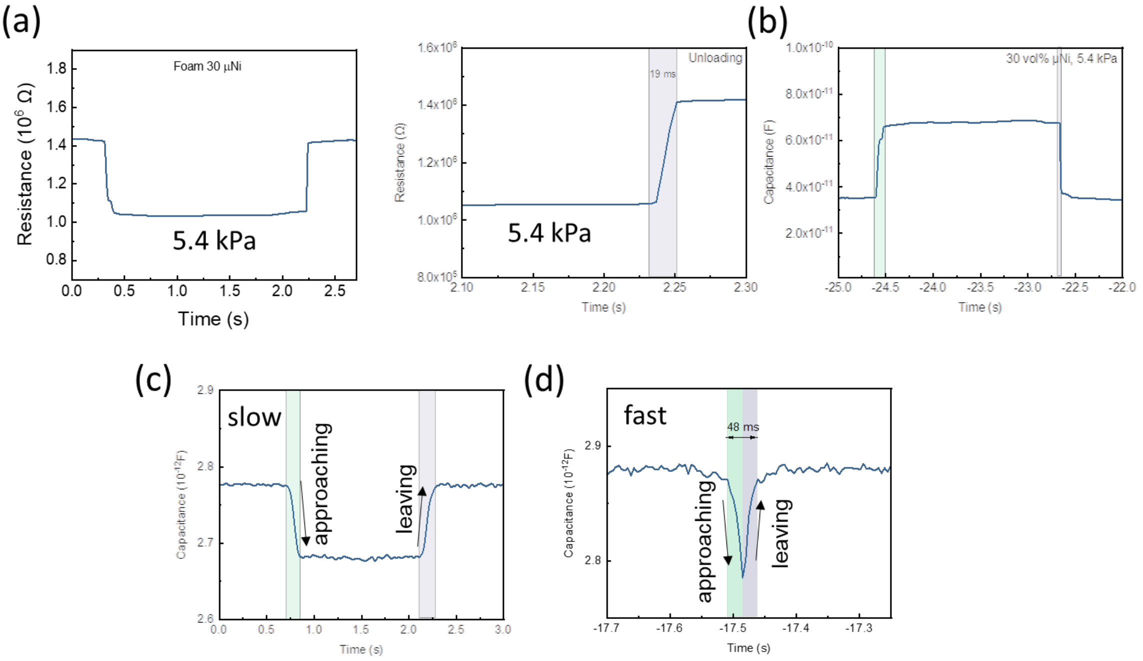

Figure 23 Sensor response time of AiFoam 30μNi. a, Pressure sensing response time in terms of piezoresistance. The right figure shows the magnified curve of the left one. The response time was as fast as 33 ms upon loading of 5.4 kPa, while the response time upon releasing of the load was 19 ms. The sensor response time is comparable to the previous studies[7–9]. **b,** Pressure sensing response time in terms of piezocapacitive. Note that the test results were obtained simultaneously with piezoresistance in **a**. **c,** The response time for AiFoam as proximity sensor is limited by the tested object moving rate. When human hand approached the sensor with slow rate, the response time was 124 ms. **d,** For fast moving object, the response time can reach 24 ms.

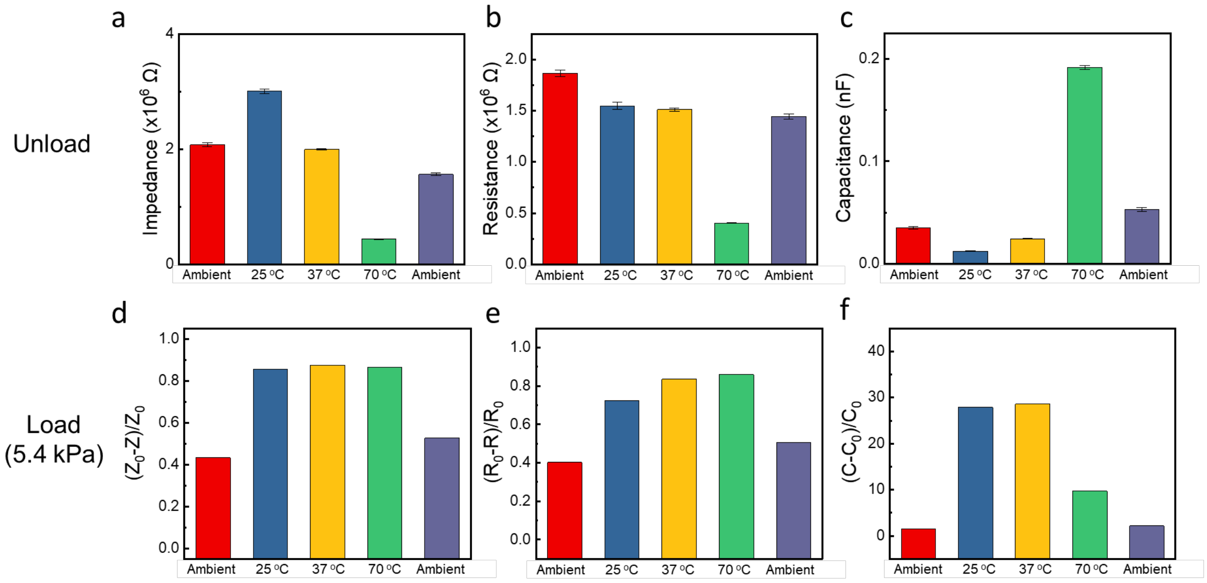

Figure 24 AiFoam 30 μNi sensor responses to temperature. Both impedance (**a-c,** absolute impedance, resistance and capacitance) and impedance change (**d-f,** change in absolute impedance, resistance and capacitance) shows response to temperature (constant relative humidity at 60 %). Ambient represents an indoor air-conditioned environment with the temperature of ~23 °C with relative humidity of ~50 %. The error bar represents standard deviations for a continuous 1-minute measurement from LCR meter with a sampling rate of 200 per sec. As the temperature increased, resistance decreased whereas capacitance increased. The absolute impedance (combination of resistance and capacitance) decreased. However, the impedance change shows the opposite behavior. The resistance increased moderately while capacitance decreased. The absolute impedance change increased from ambient environment to 25 °C then barely changed. Both impedance and impedance change can recover to its initial state after being brought back to the ambient environment.

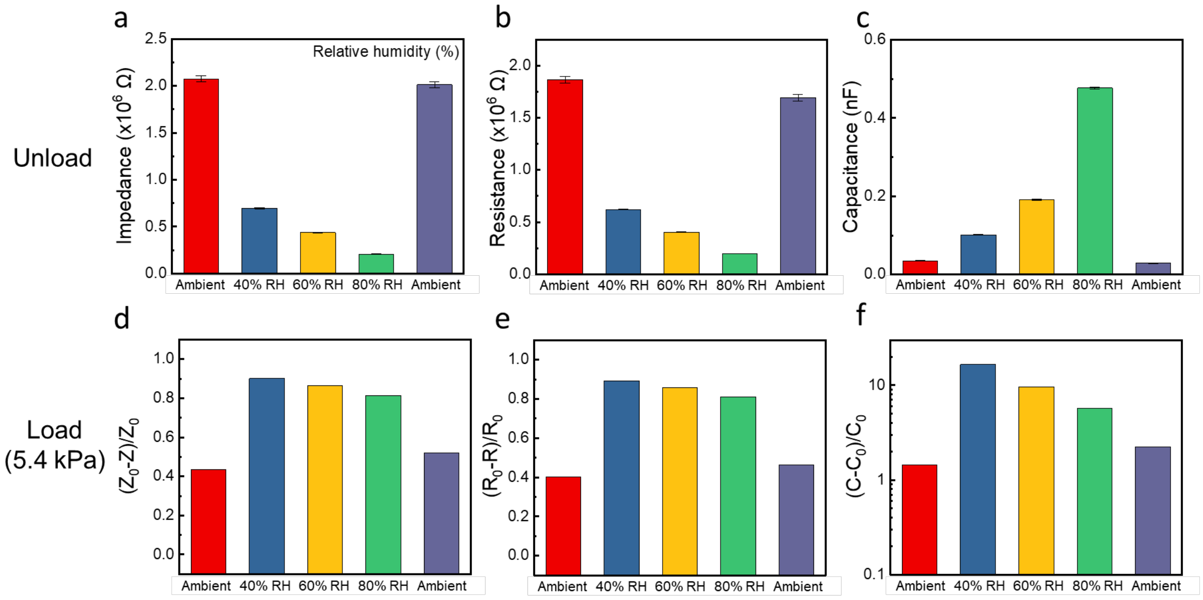

Figure 25 AiFoam 30 µNi responses to humidity. Both impedance (**a-c,** absolute impedance, resistance and capacitance) and impedance change (**d-f,** change of absolute impedance, resistance and capacitance) shows response to humidity (temperature maintained at 70 °C at all the tested humidity levels). Ambient represents an indoor air-conditioned environment with the temperature of ~23 °C with relative humidity of ~50 %. The error bar represents standard deviations for a continuous 1-minute measurement from LCR meter with a sampling rate of 200 per sec. Resistance and resistance change upon loading decreased whereas capacitance and capacitance change shows the opposite behaviour. The absolute impedance shows the similar trend as the resistance. Both impedance and impedance change can recover to its initial state at ambient environment.

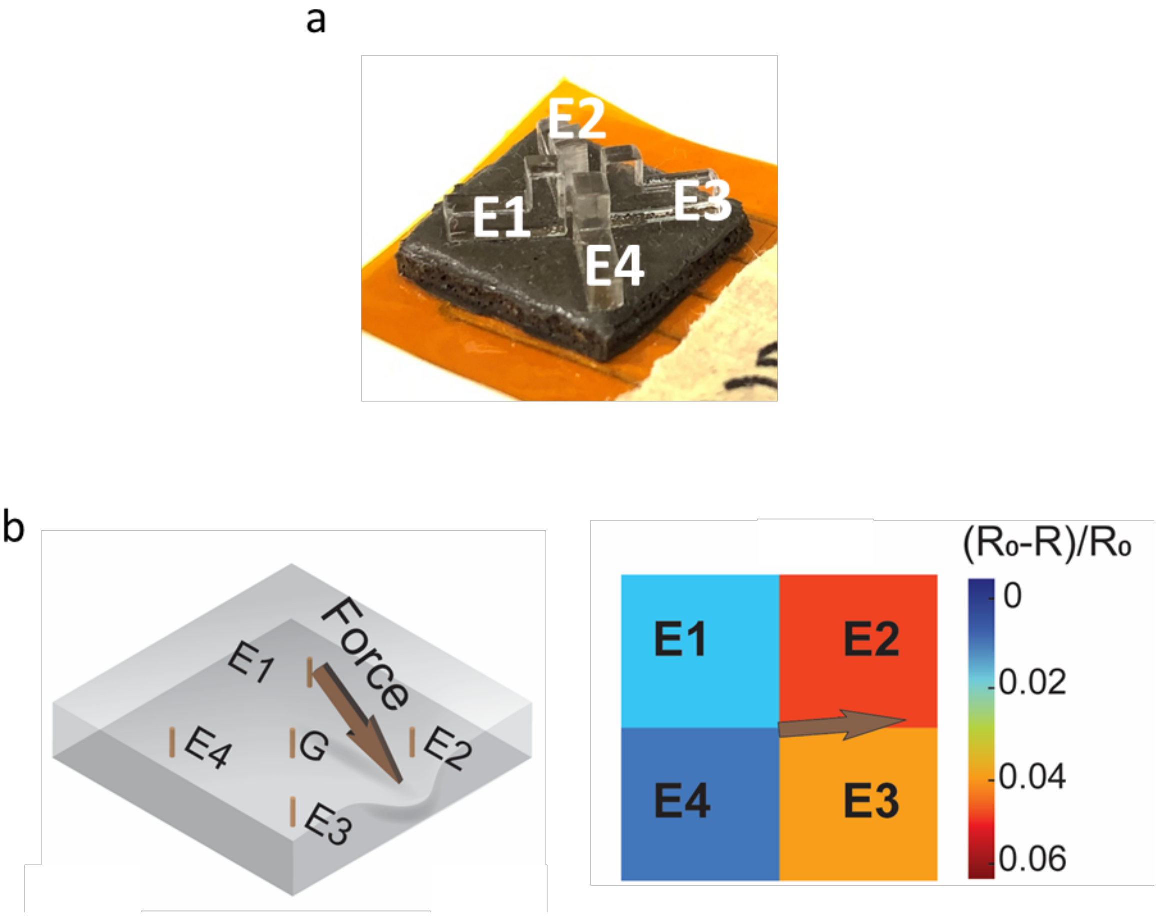

Figure 26 a, Photograph of AiFoam sensor with 4 zones for force distribution and direction identification test. **b,** AiFoam sensor (25 vol% μNi) in detecting the direction and force distribution. **Left,** Illustration of the deformation of a pixelated AiFoam sensor under external force loading**; right,** Resistance change response to the external force loadings. E1-E4 represents the 4 working electrodes and G represents the ground electrode. R_0 represents the initial resistance of the sensor and R represents the resistance of the sensor during the pressing test.

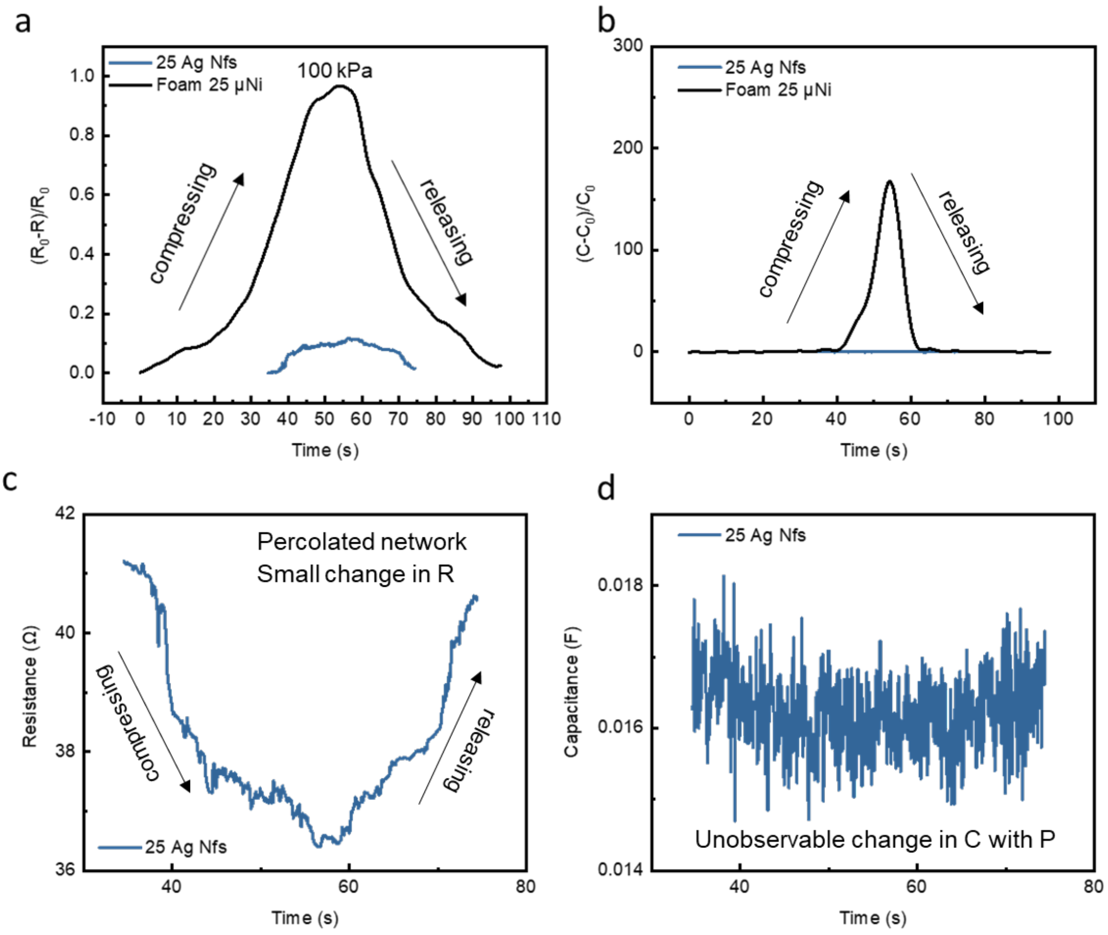

Figure 27 a, Resistance change response of material with 25 vol% Ag nanoflakes (Ag Nfs) and 25 vol% μNi under 100 kPa. Samples with Ag Nfs were not able to generate consistent foams like the samples with μNi. **b,** Capacitance change response of material with 25 vol% Ag nanoflakes and 25 vol% μNi under 100 kPa. **c,** Resistance response of material with 25 vol% Ag Nfs under 100 kPa. **d,** Capacitance response of material with 25 vol% Ag Nfs under 100 kPa. The material with 25 vol% Ag Nfs shows much smaller resistance and capacitance change than Foam 25 μNi, mainly due to the low resistance of the 25 Ag Nfs.

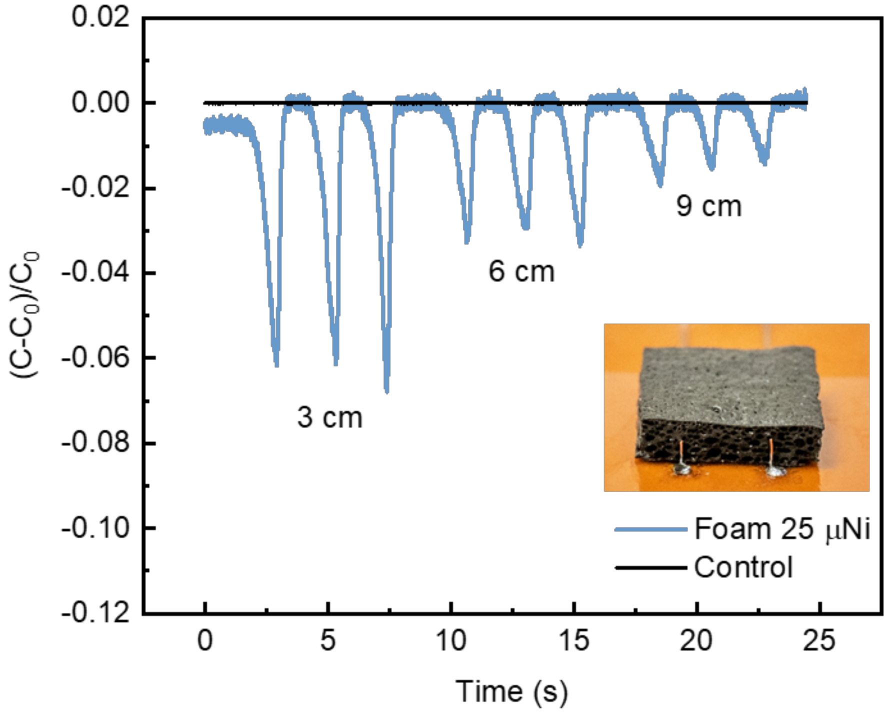

Figure 28 Proximity test of the AiFoam sensor. **Inset:** cross-section of AiFoam sensor (3D electrodes was buried inside the Foam 25 μNi); Graph shows the capacitance change response on proximity test. During the test, a human finger will approach the sensor with a distance of 3 cm, 6 cm, 9 cm, respectively. The control sample (with very low resistivity) shows no proximity performance.

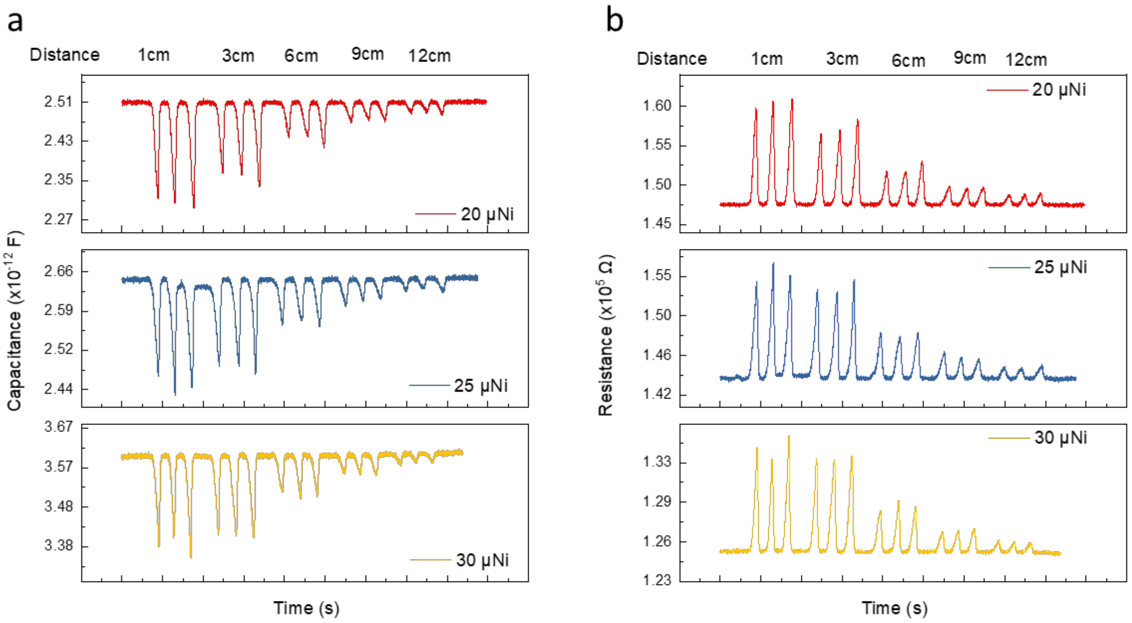

Figure 29 a, Capacitance response of AiFoam sensor as a proximity sensor. **b,** Resistance response of the AiFoam sensor as a proximity sensor. Capacitance decreased while resistance increased when the AiFoam sensor worked as a proximity sensor. An increase in µNi loading led to an increase in initial capacitance and a decrease in initial resistance.

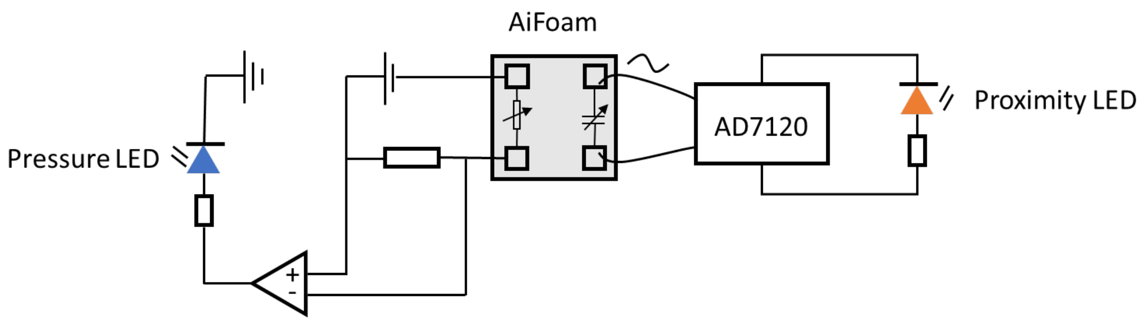

Figure 30 Electrical circuit for proximity and pressure LED demonstration.

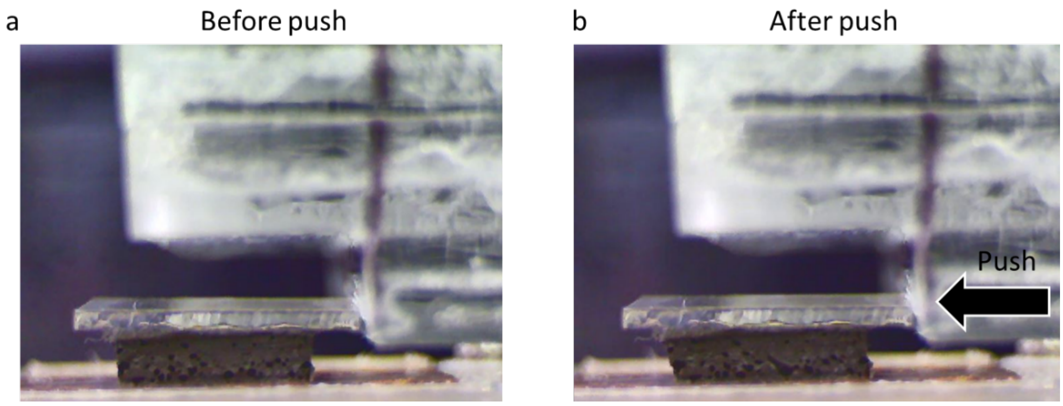

Figure 31 Shear force test of AiFoam sensor. **a,** before shear force applied. **b,** after the shear force applied. A glass slide with a bigger surface area than the top surface of the foam material adhered to the top of the AiFoam sensor. An x-y stage was employed to push the glass slide horizontally and therefore applied a shear force to the AiFoam sensor.

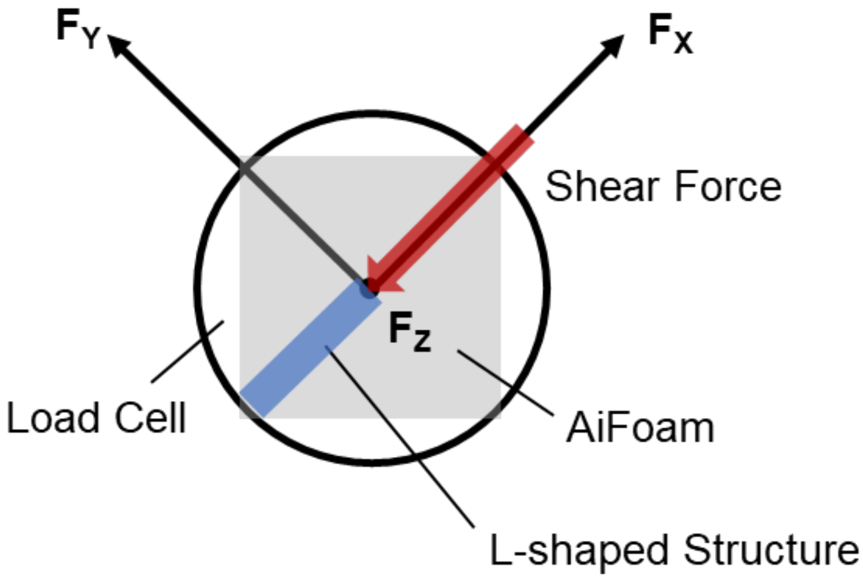

Figure 32 Calibration setup for force vector estimation.

Supplementary Tables

Table 1 Self-healing efficiency (toughness) of the base elastomer.

Fluorosurfactant concentration (wt%)	Self-healing conditions	Self-healing Efficiency (%)
30	Room temperature, 1d	2.3
50	Room temperature, 1d	7.7
70	Room temperature, 1d	5.1
50	70 ^{O}C, 1d	37.7 ± 20
50	70 ^{O}C, 4d	63.2 ± 14.6
50	70 ^{O}C, 7d	73.5 ± 10.2

Table 2 Young's moduli of Foam materials and base elastomer

Sample	Modulus (MPa)
Base elastomer	0.31
Foam 20 µNi	0.60
Foam 25 µNi	0.79
Foam 30 µNi	1.1

Table 3. Parameters of elements for samples with different Ni concentrations.

	20 vol% Ni	25 vol% Ni	30 vol% Ni	35 vol% Ni
R_1 (Ohm)	6256	3791	3307	2146
CPE_1 (S s^n)	3.3887E-11	6.0444E-11	8.1666E-11	1.8482E-10
n_1	0.91369	0.90395	0.91183	0.88672
R_2 (Ohm)	4.6281E5	3.321E5	1.3841E5	8.0993E4
W-R	87855	1.1448E5	1.6197E6	9.4493E5
W-T	0.38396	0.35555	12.67	9.495
W-P	0.59099	0.59497	0.28424	0.75116
CPE_2 (S s^n)	8.4519E-08	1.1184E-07	1.9044E-07	3.8123E-07
n_2	0.58789	0.55675	0.57786	0.49675
R_3 (Ohm)	1.635E6	2.2326E6	1.5975E6	1.1391E6
χ^2	1.756E-4	1.58E-4	1.33E-4	2.14E-4

Table 4. Parameters of elements for samples with different Ni concentrations without pores.

	20 vol% Ni	25 vol% Ni	30 vol% Ni	35 vol% Ni
R_1 (Ohm)	1233	910.8	777.2	791.1
CPE_1 (S s^n)	1.08E-10	1.7741E-10	2.0625E-10	4.0697E-10
n_1	0.91137	0.89459	0.90614	0.8765
R_2 (Ohm)	97742	75862	48633	41950
W-R	1.2431E7	2.2722E6	2.5736E6	4.5242E6
W-T	61.02	20.39	25.24	59.57
W-P	0.98187	0.92403	0.89463	0.9859
CPE_2 (S s^n)	4.2263E-7	3.6784E-7	6.5116E-7	6.3544E-7
n_2	0.44413	0.50867	0.45999	0.51816
R_3 (Ohm)	4.7431E5	4.183E5	3.67E5	6.6058E5
χ^2	2.5343E-4	4.16E-4	1.3286E-4	1.3436E-4

Table 5. Parameters of elements for samples with different Zonyl concentrations.

	No Ni	40% Zonyl	50% Zonyl	70% Zonyl
R_1 (Ohm)	263.9	199.2	554.8	769.6
CPE_1 (S s^n)	2.6434E-10	1.8065E-11	3.3271E-11	3.2627E-11
n_1	0.87024	0.9111	0.90074	0.90907
R_2 (Ohm)	61178	3.657E6	6.0732E5	2.91E5
W-R	8.3359E5	2.6046E7	2.5514E6	84152
W-T	10.78	23.846	4.505	0.081854
W-P	0.82734	0.23815	0.25977	0.46964
CPE_2 (S s^n)	4.6677E-6	1.518E-8	3.5834E-8	6.8153E-8
n_2	0.88823	0.69512	0.69872	0.56845
R_3 (Ohm)	1.7432E5	1.4315E7	2.0725E6	1.0655E6
χ^2	3.83E-4	4.91E-4	3.84E-4	7.19E-4

Table 6. μNi concentrations.

Vol% relative to P(VDF-HFP)	Wt% relative to AiFoam composite	Vol% relative to AiFoam composite	Density of AiFoam composite (g/cm^3)
20	38.20	7.580	1.766
25	45.18	10.31	2.030
30	51.44	12.73	2.202
35	57.10	14.53	2.264

Supplementary References

1. Zhou, R. *et al*. Chemically cross-linked ultrathin electrospun poly(vinylidene fluoride-co-hexafluoropropylene) nanofibrous mats as ionic liquid host in electrochromic devices. *Polymer (Guildf)*. **55**, 1520–1526 (2014).

2. Sim, L. N., Majid, S. R. & Arof, A. K. FTIR studies of PEMA/PVdF-HFP blend polymer electrolyte system incorporated with LiCF 3SO 3 salt. *Vib. Spectrosc.* **58**, 57–66 (2012).

3. Tian, X. & Jiang, X. Poly(vinylidene fluoride-co-hexafluoropropene) (PVDF-HFP) membranes for ethyl acetate removal from water. *J. Hazard. Mater.* **153**, 128–135 (2008).

4. Gnana Kumar, G. Irradiated PVdF-HFP-montmorillonite composite membranes for the application of direct ethanol fuel cells. *J. Mater. Chem.* **21**, 17382–17391 (2011).

5. Wu, C. M., Chou, M. H. & Zeng, W. Y. Piezoelectric response of aligned electrospun polyvinylidene fluoride/carbon nanotube nanofibrous membranes. *Nanomaterials* **8**, 1–13 (2018).

6. Tung, T. T. *et al*. Enhancing the sensitivity of graphene/polyurethane nanocomposite flexible piezo-resistive pressure sensors with magnetite nano-spacers. *Carbon N. Y.* **108**, 450–460 (2016).

7. Lee, J. *et al*. Conductive fiber-based ultrasensitive textile pressure sensor for wearable electronics. *Adv. Mater.* **27**, 2433–2439 (2015).

8. Pan, L. *et al*. An ultra-sensitive resistive pressure sensor based on hollow-sphere microstructure induced elasticity in conducting polymer film. *Nat. Commun.* **5**, 1–8 (2014).

9. Lv, G. *et al*. Flexible, Conformable Organic Semiconductor Proximity Sensor Array for Electronic Skin. *Adv. Mater. Interfaces* **2000306**, 1–7 (2020).
