# Supplementary material for: Artificially innervated self-healing foams as synthetic piezo-impedance sensor skins
Source: Nat Commun. 2020 Nov 12;11:5747. doi: 10.1038/s41467-020-19531-0 (PMC7665015; doi:10.1038/s41467-020-19531-0)
Supplement: Supplementary file 2 — Description of Additional Supplementary Files [file 41467_2020_19531_MOESM2_ESM.pdf]

Description of Additional Supplementary Files

File Name: Supplementary Movie 1

Description: Stretchability and self-healing ability of AiFoam elastomer Stretchability and self-healing ability of the base elastomer material for AiFoam. The video first shows the high stretchability of the elastomer. The later part of the video shows the restoration of the stretchability of the elastomer after bifurcation and self-healing at 70 o C for 4 days.

File Name: Supplementary Movie 2

Description: Direction detection The AiFoam sensor with five 3D electrodes, i.e. four working electrodes and one ground electrode, is used for direction detection. An external force is applied to the sensor. The inset pressure map shows the resistance change caused by the force, indicating the direction of the applied force.

File Name: Supplementary Movie 3

Description: Proximity sensing An orange LED connected to the AiFoam sensor and an external circuit (Supplementary Fig. 30) is activated when the human finger approaches to the sensor.

File Name Supplementary Movie 4

Description: Proximity and pressure sensing Proximity and pressure sensing of AiFoam are indicated by an orange LED and a blue LED, respectively. The two LEDs are connected to the AiFoam through an external circuit (Supplementary Fig. 30). The blue LED is activated, indicating the electrical circuit is turned on. Then the orange LED lighted up when the human finger approaches to the sensor, and the blue LED becomes brighter when the finger touches and presses the sensor.
